# Exact approaches for scaffolding

**DOI:** 10.1186/1471-2105-16-S14-S2

**Published:** 2015-10-02

**Authors:** Mathias Weller, Annie Chateau, Rodolphe Giroudeau

**Affiliations:** 1 Laboratoire d'Informatique, de Robotique et de Microélectronique de Montpellier (LIRMM) - Université de Montpellier - UMR 5506 CNRS, 161 rue Ada, 34090 Montpellier, France; 2 Institut de Biologie Computationnelle, Lirmm Bât 5 - 860 rue de St Priest, 34090 Montpellier, France

**Keywords:** scaffolding, exact methods, dynamic programming, treewidth, preprocessing, fixed-parameter tractable

## Abstract

This paper presents new structural and algorithmic results around the scaffolding problem, which occurs prominently in next generation sequencing. The problem can be formalized as an optimization problem on a special graph, the "scaffold graph". We prove that the problem is polynomial if this graph is a tree by providing a dynamic programming algorithm for this case. This algorithm serves as a basis to deduce an exact algorithm for general graphs using a tree decomposition of the input. We explore other structural parameters, proving a linear-size problem kernel with respect to the size of a feedback-edge set on a restricted version of Scaffolding. Finally, we examine some parameters of scaffold graphs, which are based on real-world genomes, revealing that the feedback edge set is significantly smaller than the input size.

## Introduction

During the last decade, a huge amount of new genomes have been sequenced, leading to an abundance of available DNA resources. Nevertheless, most of recent genome projects stay unfinished, in the sense that databases contain much more incompletely assembled genomes than whole stable reference genomes [1]. One reason for this phenomenon is that, for most of the analyses performed on DNA, an incomplete assembly is sufficient. Sometimes it is even possible to perform them directly from the sequenced data. Another reason is that producing a complete genome, or an as-complete-as-possible-genome is a difficult task. Traditionally, producing a complete genome consists of three steps, each of them computationally or financially difficult: the assembly, the scaffolding, and the finishing. The step of scaffolding, on which we focus here, consists of orienting and ordering at the same time the contigs produced by assembly. Many methods have been proposed and the recent activity on the subject shows that it is an active field (see, not exhaustively, [2-8] and Section ). A good survey of recent methods can be found in [9] for instance. Since the problem has been proved NP-complete from its first formulation [10], nearly all of these methods propose heuristic approaches. One of them claims that an exact method provides better results [5], however, the authors prepend a heuristic graph simplification before running their exact algorithm.

The approach presented here relies on a combinatorial problem on a dedicated graph, called *sca*ff*old graph*, representing the link between already assembled contigs. The main idea is to represent each contig by two vertices linked by an edge (these "contig-edges" form a perfect matching on the scaffold graph). Other edges are constructed and weighted using complementary information on the contigs. The weight of a non-contig edge *uv*, with *uu′, vv′ *being contig-edges, corresponds to a confidence measure that the *uu′ *contig is succeeded by the *vv′ *contig (oriented as *u′ *− *u *− *v *− *v′*). The scaffold graph is a flexible tool to study the scaffolding issues. Indeed, the graph is a syntactical data-structure which may represent several semantics. For instance, the scaffold graphs used for our previous experiments have been built using Next-Generation Sequencing data, namely paired-end reads. However, we also could provide other type of information to compute the weight on the edges, like for instance ancestral support in a phylogenetic context, or comparison to other extent genomes which could be used as multiple references. The way to define the weight on the edges does not change the main goal of our method, which is to determine the optimal ordering and orientation of the contigs, given a specific criterion. It is also possible to mix two or more criteria in order to take several sources of information into account.

We also introduced two structural parameters *σ*_p _and *σ*_c _representing the respective numbers of linear and circular chromosomes sought in the genome. Those parameters seems quite artificial at a first sight, but they are well-motivated as follows. First, in a number of species, genomes are hard to assemble with classical methods because of an heterogeneous chromosomal structure or difficulties to perform classical assembly. This is the case, for instance, with the micro-chromosomes in the chicken genome [11]. Also, when scaffolding metagenomic data, one has to deal with very complex genomic structures [12]. We hope that relaxing the classical model with one chromosome, mixed with the flexibility of the scaffold graph, could help handling such complex situations. The second motivation to introduce those parameters came from the desire to explore an intermediary case between the very classical and studied NP-complete [13]TRAVELING SALESMAN PROBLEM (TSP) and a totally free structure, in which case optimizing a covering by paths and cycles is polynomial-time solvable [14]. The SCAFFOLDING problem then consists of finding (at most) *σ*_p _paths and *σ*_c _cycles that cover all contig-edges while maximizing the total weight. Previously, we proved that SCAFFOLDING is NP-complete, even under restricted conditions [15], developed polynomial-time approximation algorithms [15,16] and evaluated them experimentally [17]. In this paper, we continue to explore this problem, as well as the structural properties of the scaffold graph. This exploration aims to develop an efficient method, that could be used both to refine the ratio analysis on previously designed heuristics and to handle cases of small genomes with better quality.

We present both theoretical and practical results, as well as our experimental findings. The paper is organized as follows: we relate a general state-of-the-art and methodological comparison with existing methods and our previous work in Section. In Section, we formally introduce the problem and some notations and definitions. In Section, we show that the general SCAFFOLDING problem is polynomial-time solvable on trees, and present a dynamic programming algorithm. We extended this algorithm in Section 11 to a *parameterized *algorithm with respect to the *treewidth *of the input, that is, this algorithm runs in polynomial time, on scaffold graphs that are sparse, *i.e*. treelike. In Section 11, we lay the foundation to developing effective preprocessing routines for SCAFFOLDING, by presenting a set of reduction rules whose application shrinks input instances of a derived problem, RESTRICTED SCAFFOLDING. We show that the size of the resulting instances can be bounded linearly in their feedback edge set number, thus proving a problem kernel for this parameter. Finally, in Section 11, we present some experimental analysis of scaffold graphs that are derived from real-world genomes.

## Related work

Genome scaffolding is an intrinsically complex problem, and was studied through several computation models and tools. The first model, presented in [10], leads to its classification as an NP-complete problem. This work also proposes the first greedy approach. The scaffolding was then performed using genomic fragments coupled by mate-pairs [18], which differ from the paired-end reads essentially by their larger insert-size (The insert-size is the gap, in base pairs, between two fragments constituting a pair of reads), and their lower covering depth, so the size of the data and the organization of the graph may differ from actual Next-Generation Sequencing data using paired-end reads. A greedy-like approach is also used in SSPACE [19], iteratively combining the longest contigs. Conversely, Gao et al. present a dynamic-programming-based exact approach (OPERA) which provides higher-quality scaffolds than existing heuristic approaches [5]. However, their algorithm runs in *O*(*n^k ^*) time (where *k *is the "library width") which quickly grows out of reasonable proportions. Thus, to apply this method on real data, they prepend a graph contraction procedure to limit the size of the input. In SOPRA [4], Dayarian et al. introduce a removal procedure for problematic contigs, and separate the orientation step from the linking step. The orientation step uses a simulated annealing technique for regions of high complexity. In GRASS [3], a genetic algorithm is provided, using mixed integer programming (MIP) as in [6] and an expectation-maximization process to counter the intractability of the MIP model. They obtain results which are intermediary in quality between SSPACE and Opera. In SCARPA [2], the two problems of orienting and ordering the contigs are separated, as in SOPRA. Transforming the graph such that the contig orientation problem has a feasible solution is a fixed-parameter tractable problem with respect to the number of nodes to remove [20]. The authors first use the corresponding algorithm to pre-process the graph and exhibit an optimal relative orientation of the contigs. Then, pre-oriented contigs are ordered using a heuristic, and the removal of articulation vertices is used to limit the size of the connected components. Misassembled contigs are detected and removed. In BESST [21], the authors refine the previous approaches by considering the library insert size distribution to filter relevant linking information, leading to an almost linear scaffold graph, which is easy to treat. The scalability of these approaches is not always proven, and the general increase of the size and amount of available data brings real efficiency questions. Recently, some methods also use phylogenetic or comparative genomic approaches via rearrangement analysis to perform scaffolding on ancient or extant genomes when a set of closely related species genomes are available (see for instance [22-24]). This encourages the mix of multiple sources of information for scaffolding a given genome.

The scaffolding problem have been extensively studied in the framework of complexity and approximation. In [15,16], the authors proved that the problem is NP-complete even if the scaffold graph is a planar bipartite graph. They also proposed some lower bounds for exact exponential-time algorithms for SCAFFOLDING according to the EXPONENTIAL-TIME HYPOTHESIS[25]. Finally, they proved that the minimization version of SCAFFOLDING (seeking a *minimum-weight *cover of the scaffold graph) is unlikely to be approximable within any constant ratio, even if the scaffold graph is a completed bipartite graph [15]. On the positive side, two polynomial-time factor-3-approximation algorithms for the maximization version have been designed. The first is an *O*(*n*^2 ^log *n*)-time greedy algorithm, the second is based on the computation of a maximum matching (*O*(*n*^3^)-time). The theoretical aspects of the scaffold graph have been completed by extensive experimental results [17] for the maximization version on simulated and real datasets.

The main contribution of the present paper lays in both the algorithmic exploration of exact methods for the scaffolding problem with structural parameters, and the initiation of a discussion on the scaffold graph properties. Indeed, the knowledge of those properties may lead to algorithmic improvement, as well as the detection of putative errors in assemblies.

## Definitions

*Sca*ff*olding problem*. The central combinatorial object we are working with in this work is the "scaffold graph" (see also [16,17]).

**Definition 1 **(Scaffold graph) *A *scaffold graph *is a pair *(*G, M*) *of a graph G *= (*V, E, w*) *with an even number of vertices, and a weight function w *: *E *→ ℕ *on its edges, and a perfect matching M on G*.

**Figure 1:**
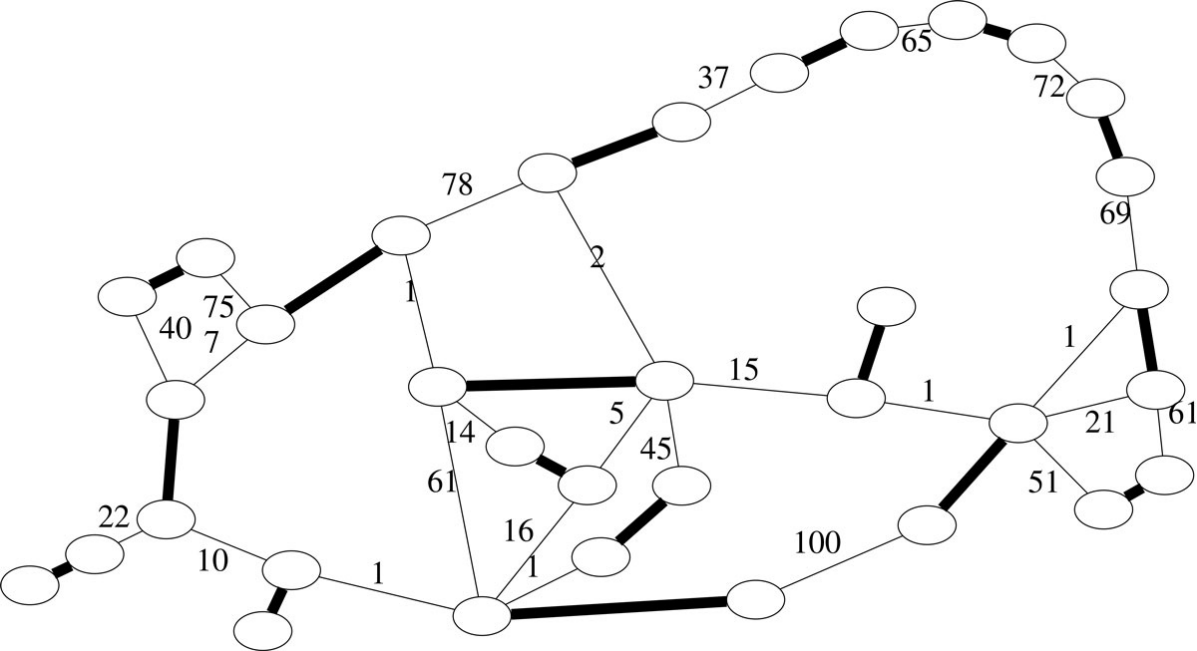
**A scaffold graph with 17 contigs (bold edges) and 26 (weighted) links between them, corresponding to the ebola data set (see Section 11)**.

Notice that this model is close to what previous work call scaffold graph, except that this graph is not a directed graph, and contigs are represented by edges instead of vertices. Figure 1 shows an example of a scaffold graph.

*Graph Theory*. Slightly abusing notation, we sometimes consider paths as sets of edges. Furthermore, for a matching *M *and a vertex *u*, we define *M*(*u*) as the unique vertex *v *with *uv *∈ *M *if such a *v *exists, and *M*(*u*) = ⊥, otherwise. A path *p *is *alternating *with respect to a matching *M *if, for all vertices *u *of *p*, also *M*(*u*) is a vertex of *p*. If *M *is clear from context, we do not mention it explicitly. For a graph *G *= (*V, E*) and a vertex set *V*^′ ^⊆ *V*, let *G*[*V*^′^] denote the subgraph of *G *induced by *V*^′ ^and let *G *− *V*^′ ^:= *G*[*V *\ *V*^′^]. For *S *⊆ *E*, let *G *− *S *:= (*V, E *\ *S *) and, for any edge or vertex *x*, we abbreviate *G *− {*x*} =: *G *− *x*. For a set of pairs *S *, we let Gr(*S *) denote the graph having *S *as edgeset, that is, Gr(*S *) := (U*_e∈S _*e, S ). For a function ω : *E *→ ℕ and a set *S *⊆ *E*, we abbreviate Σ*_e∈S _*ω(*e*) =: *ω*(*S *). Let *G *= (*V, E*) be a graph with a matching *M *and let *S *be a matching in *G *− *M*. The number of paths (resp. cycles) in *G*[*S *∪ *M*] is denoted by $||S|{|}_{p}^{G,M}$ (resp. $||S|{|}_{c}^{G,M}$). If all paths in *G*[*S *∪ *M*] are alternating with respect to *M*, then, we call *S *a ||*S*||*_p_-*||*S*||*_c_-cover *(or simply *cover*) for *G *with respect to *M *(we will omit *M *if its clear from context). If ||*S*||*_c _*= 0, we may also refer to *S *as a ||*S*||*_p_-path cover *(or simply *path cover*). The general scaffold problem is expressed as follows (see also [16,17]): SCAFFOLDING (SCA)

**Input: **A scaffold graph (*G *= (*V, E, w*), *M*), *σ*_p _∈ ℕ, *σ*_c _∈ ℕ, *k *∈ ℕ

**Question: **Is there a *σ*_p_-*σ*_c_-cover *S *for *G *with respect to *M *with *ω*(*S *) ≥ *k*?

*Tree Decompositions*. A tree decomposition of a graph *G *= (*V, E*) is a pair (*T *= (*V^T ^, E^T ^*), *X *: *V^T ^*→ 2*^V ^*) such that (1) for all *uv *∈ *E*, there is some *i *∈ *V^T ^*with *uv *⊆ *X*(*i*) and (2) for all *v *∈ *V*, the subtree *T_v _*:= *T*[{*X*(*i*) | *v *∈ *X*(*i*)}] is connected. We call the images of *X *"bags" and the size of the largest bag minus one is the *width *of the decomposition. A decomposition of minimum width for *G *is called the *optimal *for *G *and its width is called the *treewidth *of *G*. It is a folklore theorem that each graph *G *has an optimal tree decomposition (*T, X*) that is *nice*, that is, each bag *X*(*i*) is one of the following types:

**Leaf bag: ***i *is a leaf of *T *and *X*(*i*) = ∅,

**Introduce vertex ***v ***bag: ***i *is internal with child *j *and *X*(*i*) = *X*( *j*) ∪ {*v*} with *v *∉ *X*(*j*),

**Forget ***v ***bag: ***i *is internal with child *j *and *X*(*i*) = *X*(*j*) − *v *with *v *∈ *X*(*i*),

**Introduce edge ***uv ***bag: ***i *is internal with child *j *and *uv *∈ *E *and *uv *⊆ *X*(*i*) = *X*(*j*),

**Join bag: ***i *is internal with children *j *and ℓ and *X*(*i*) = *X*(*j*) = *X*(ℓ).

Additionally, each edge *e *∈ *E *is introduced exactly once. Given a width-tw tree decomposition, we can obtain a nice tree decomposition of width tw in *n *· poly(tw) time [26].

*Parameterized Algorithmics*. Parameterized complexity theory challenges the traditional view of measuring the running times exclusively in the input size *n*. Instead, we aim at identifying a parameter of the input that we expect to be small (much smaller than *n*) in all instances that the application at hand may produce. We then focus on developing algorithms whose exponential part can be bounded in this parameter (see [27] for details). Parameterized complexity allows to prove performance guarantees for preprocessing algorithms: a polynomial-time algorithm that, given an instance *x *with parameter *k*, computes an instance *x′ *with parameter *k′ *≤ *k *such that *x *is a yes instance if and only if *x′ *is a yes instance, is called *kernelization *and the result *x′ *is the *kernel*. It is common to present a kernelization by showing various *reduction rules *that "cut away" the easy parts of the input and, when applied exhaustively to the input, shrink it enough to prove the size bounds.

## Scaffolding on trees

Towards developing an algorithm for SCAFFOLDING that runs fast on graphs that are "close to trees", we consider a strategy to solve SCAFFOLDING in case the input graph is a tree. We use a bottom-up dynamic programming approach that computes for each vertex *v *starting with the leaves, the best possible solutions for the subtree rooted at *v*. For ease of presentation, we thus consider the input tree *T *to be rooted arbitrarily with *r *denoting the root vertex. Note that the solution cannot contain any cycles in this case.

At each vertex, the dynamic programming algorithm needs to decide how many paths should be covered in each of the subtrees of its children. Seeing that it is infeasible to try all combinations, we employ, again, a dynamic programming strategy solving this problem for a given vertex *v*: We order the children of each vertex *v *arbitrarily and let *s_v _*denote the sequence of children of *v *with *s_v_*[*j*] denoting the *j*^th ^child of *v *and *s_v_*[1..*j*] := U*_i≤j _s_v_*[ *j*]. Then, we update the global dynamic programming table of *v *in order of *s_v_*. Thus, when we consider the *j*^th ^child *u *of *v*, we only have to split the paths to be covered between the two subtrees *T_u _*and the union of all previously considered subtrees rooted at children of *v *(that is, *T *[U_ℓ<*j *_*T_sv [ℓ] _*∪ {*v*}]).

In the following, for a vertex *v*, let *C*(*v*) denote its children and par(*v*) its parent (or ⊥ if *v *= *r*), and let *T_v _*denote the subgraph of *T *that is rooted at *v*. For *u, v *∈ *V*, we define *T_u _*+*T_v _*:= *T *[*V*(*T_u_*)∪*V*(*T_v_*)] and *T_u _*+*v *:= *T *[*V*(*T_u_*)∪{*v*}]. Let *v *∈ *V *and 1 ≤ *j *≤ |*C*(*v*)|. Then, we define $T_{j}^{v}:=\sum_{i\leq j}T_{s_{v}\left[i\right]}+v$. Finally, we abbreviate *s_v_* := *s_v_*[1..0] := ⊥.

**Algorithm 1: **Algorithm to fill the dynamic programming table.

**1***I *← leaves of *T*;

**2 while **∃*v *∈ *V *\ *I s.t. C*(*v*) ⊆ *I ***do**

**3     foreach **1 ≤ *j *≤ |*C*(*v*)| *with u *:= *s_v_*[*j*] **do**

**4         foreach ***i *≤ *σ*_p _**do**

**5             if ***uv *∈ *M ***then**

**6 **                [0, *i, j*]*_v _*← max_α∈{0,1} _max_ℓ≤*i*−(1−α)_[α, ℓ, ∞]*_u _*+ [0, *i *− (ℓ − α + 1), *j *− 1]*_v_*;

**7 **                [1, *i, j*]*_v _*← max_α∈{0,1} _max_ℓ≤*i*_[α, ℓ, ∞]*_u _*+ [1, *i *− (ℓ − α), *j *− 1]*_v_*;

8             else

**9 **                [0, *i, j*]*_v _*← max_ℓ≤*i *_max_α∈{0,1}_[α, ℓ, ∞]*_u _*+ [0, *i *− ℓ, *j *− 1]*_v_*;

**10**               ${\left[\text{1},i,j\right]}_{v}\leftarrow \underset{\ell \leq i}{\text{max}}\left\{\begin{array}{c} \text{ma}{\text{x}}_{\alpha \in \left\{0,\text{1}\right\}}{\left[\alpha ,\ell ,\infty \right]}_{u}+{\left[\text{1},i-\ell ,j-\text{1}\right]}_{v};\\ \omega \left(uv\right)+{\left[0,\ell ,\infty \right]}_{u}+\left\{\begin{array}{c} {\left[0,i-\left(\ell -\text{1}\right),j-\text{1}\right]}_{v}\;\;\;\;\;\;\;\;\;;\text{if}\;w\in s_{v}\left[\text{1}..j\right]\\ {\left[0,i-\ell ,j-\text{1}\right]}_{v}\;\;\;\;\;\;\;\;\;\;\;\;\;\;\;\;\;\text{otherwise} \end{array}\right) \end{array}\right)$

**11 if ***v *= *r ***then return **max*_c∈{0,1}_*[*c, σ*_p_, ∞]*_r_***else*** I *← *I *∪ {*v*};

**Semantics: ***For *(*c, i, j, v*) ∈ {0, 1} × [*σ*_p_] × [|*C*(*v*)|] × *V we let *[*c, i, j*]*_v _denote the maximum weight of an i-*0*-cover S for $T_{j}^{v}$ such that v is incident with exactly c edges of S. We abbreviate *[*c, i*, ∞]*_v _*:= [*c, i*, |*C*(*v*)|]*_v_*.

We maintain a set *I *of initialized vertices and, as soon as *r *is initialized, the algorithm stops. Thus, we assume *r *∉ *I*. Finally, the maximum weight of a *σ*_p_-0-cover for *T *can be computed by max*_c∈{0,1}_*[*c, σ*_p_, ∞]*_r_*.

**Lemma 1 ***Algorithm 1 is correct, that is, for all *(*i, j, c, v*) ∈ [*σ*_p_] × [|*C*(*v*)|] × {0, 1} × *V and for any maximum-weight i-*0*-cover S for $T_{j}^{v}$ with respect to M such that v is incident with exactly c edges of S we have *[*c, i, j*]*_v _*= *ω*(*S*).

Concerning the running time, we note that the body of the loop in line 3 is executed exactly once per vertex and, hence, lines 6,7,9, and 10 are executed *σ*_p _times per vertex. As they compute the maximum over *σ*_p _values, the whole algorithm runs in $O\left(n\cdot \sigma_{p}^{\text{2}}\right)$ time.

**Corollary 1 **SCAFFOLDING*on trees can be solved in*$O\left(n\cdot \sigma_{p}^{\text{2}}\right)$*time*.

## Scaffolding with respect to treewidth

**Figure 2:**
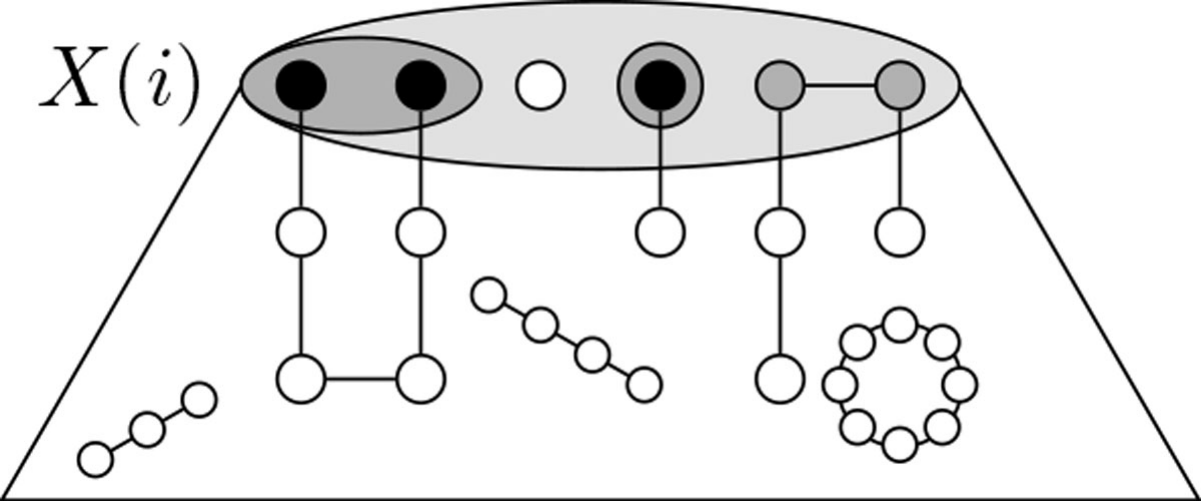
**Setup of the dynamic programming on tree decompositions**. A vertex *u *∈ *X*(*i*) can have degree *d*(*u*) = 0 (white circle), *d*(*u*) = 1 (black circle), or *d*(*u*) = 2 (gray circle) in $G_{i}^{S}$. Vertices with d(u) = 1 are always incident with paths in $G_{i}^{S}$ (gray ellipse), forming a pairing (a permutation) *P *on them.

In this section, we develop a parameterized algorithm with respect to the structural parameter "treewidth", solving SCAFFOLDING in *O*(tw^tw^) poly(*n, σ*_p_, *σ*_c_) time. It is based on using a dynamic programming table to keep track of solutions that interact with the bags of a tree decomposition in a certain way (see Figure 2). Since we store for each type of interaction only the best solution and the number of interactions can be bounded in the treewidth, we arrive at the claimed bounds.

To present our algorithm, we use special permutations (involutions) to model matchings that allow reflexive pairing (that is, matching a vertex with itself). Thus, slightly abusing notation, we will consider permutations as sets of pairs. We denote the subset of reflexive pairs of a permutation *P *by *P*^1 ^and the subset of non-reflexive pairs by *P*^2^. Then, Gr(*P*) :=Gr(*P*^2^). For permutations *P *and *Q*, we define *P *□ *Q *as the set of pairs *uv *such that *P*(*x*) = ⊥ ⊕ *Q*(*x*) = ⊥ for all *x *∈ *uv *and there is a *u*-*v*-path in Gr(*P *∪ *Q*). Furthermore, for a function *d *: *A *→ *B *and (*x, y*) ∈ *A *× *B*, we define *d*[*x *→ *y*] as the result of setting *d*(*x*) := *y *(that is (*d *\ ({*x*} × *B*)) ∪ {(*x, y*)}). Here, *d*[*x *→ ⊥] means to remove *x *from the domain of *d*. Let *T *= (χ, *E^T ^*) be a tree decomposition of *G *with root *X*(*r*) ∈ χ. For a bag *X*(*i*), let *G_i_*denote the subgraph of *G *that contains exactly those edges of *G *that are introduced in a bag of the subtree of *T *that is rooted at *X*(*i*) and let $G_{i}^{{}^{S}}:=G_{i}\left[S\cup M\right]$.

A table entry for the bag *X*(*i*) will be indexed by (i) a function *d *: *X*(*i*) → {0, 1, 2}, (ii) a permutation *P *with U*_uv∈P _uv *= *d*^−1^(1) and(iii) integers *p *≤ *σ*_p_ and *c *≤ *σ*_c_. See Figure 2 for an illustration of *d *and *P*. An entry will have the following semantics:

**Definition 2 ***Let i *∈ *V^T^. We call a set S_i _*⊆ *E*(*G_i_*) \ *M *eligible *with respect to a tuple *(*d, P, p, c, i*) *if*

*1 each vertex v *∈ *X*(*i*) *has degree d*(*v*) *in *$G_{i}^{S_{i}}$,

*2 for each uv *∈ *P, if u ≠ v, then there is a u-v-path in $G_{i}^{S_{i}}$and, if u *= *v, then there is a path q of non-zero-length in $G_{i}^{S_{i}}$ that contains u and avoids d*^−1^(1) − *u *(*we say that q is *dangling from *u*).

*3 $G_{i}^{S_{i}}$contains p paths and c cycles that do not intersect d*^−1^(1),

**Semantics: ***A table entry *[*d, P, p, c*]*_i _is the maximum weight of any set that is eligible with respect to *(*d, P, p, c, i*).

Then, we can read the maximum weight of a solution *S *for *G *from [∅, ∅, *σ*_p_, *σ*_c_]*_r_*.

Given a nice tree decomposition and a bag *X*(*i*) with children *X*(*j*) and *X*(ℓ) (possibly *j *= ℓ), we compute [*d, P, p, c*]*_i_* depending on the type of the bag *X*(*i*) (entries that are not mentioned explicitly are set to −∞):

**Leaf bag: **Set [∅, ∅, 0, 0]*_i _*:= 0.

**Introduce vertex ***v***: **Newly introduced vertices do not have introduced edges yet. Thus, we force the degree of *v *in $G_{i}^{S}$ to 0: Formally, let [*d, P, p, c*]*_i _*:= [*d*[*v *→ ⊥], *P, p, c*] *_j_*if *d*(*v*) = 0 and ∞, otherwise.

**Forget vertex ***v***: **A vertex *v *that we forget in bag *X*(*i*) can have degree 0,1, or 2 in $G_{j}^{S}$. If it has degree 1, then there is a path *q *dangling from it. If *q *ends in some other vertex *u *∈ *X*(*i*) \ {*v*} = *X*( *j*), then, the permutation for *X*(*i*) contains *uu *and the permutation for *X*(*j*) contains *uv*. Otherwise, *q *is dangling from *v *in $G_{j}^{S}$ so the permutation for *X*(*j*) contains *vv*. Formally, let

$${\left[d,P,p,c\right]}_{i}:=\text{max}\left\{\begin{array}{c} \underset{uu\in P}{\text{max}}[d{\left[v\to \text{1]},\;\left(P-uu\right)+uv,p,c\right]}_{j},\\ {}[d{\left[v\to \text{1]},P+vv,p-\text{1},c\right]}_{j},\\ \underset{x\in \left\{0,2\right\}}{\text{max}}[d[v\to x{],P,p,c]}_{j}. \end{array}\right)$$

**Introduce edge ***uv***: **Let *d*(*u*), *d*(*v*) ≥ 1 and, by symmetry, let *d*(*u*) ≥ *d*(*v*) ≥ 1. We define a value *z *(representing the assumption that *uv *is in *S *) as follows. Let *d′ *:= *d*[*u *→*d*(*u*) − 1, *v *→ *d*(*v*) − 1], that is, we let *d′ *be the result of decreasing both *d*(*u*) and *d*(*v*) by one.

**Figure 3:**
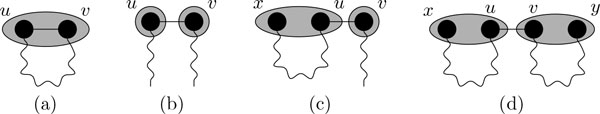
**The four cases for introducing an edge *uv *with *d*(*u*) = *d*(*v*) = 2 in the dynamic programming for *X*(*i*): (a) there is a *u*-*v*-path in $G_{j}^{S}$, (b) *u *and *v *have dangling paths in $G_{j}^{S}$, (c) there is a *u*-*x*-path and a dangling path on *v *in $G_{j}^{S}$, or (d) there is a *u*-*x*-path and a *v*-*y*-path in $G_{j}^{S}$**.

**Case 1 ***d*(*u*) = *d*(*v*) = 2. Then, *P *avoids *u *and *v*. Since we assume *uv *∈ *S *, this means that *u *and *v *have dangling paths *q_u _*and *q_v _*in $G_{i}^{S}-uv=G_{j}^{S}$ that both intersect *d′*^−1^(1). If *q_u _*= *q_v_*, then adding *uv *to *S *closes a cycle in $G_{i}^{S}$ and the permutation for *X*(*j*) contains *uv *(see Figure 3(a)). Otherwise, *q_u_*≠ *q_v_*. Then, if *q_u _*intersects *d′*^−1^(1) \ {*u, v*} in a vertex *x*, then the permutation for *X*(*j*) contains *ux *(see Figure 3(c) and Figure 3(d)), otherwise, it contains *uu *(see Figure 3(b)). Likewise, if *q_v _*intersects *d′*^−1^(1) \ {*u, v*} in a vertex *y*, then the permutation for *X*(*j*) contains *vy*, otherwise, it contains *vv*. Note that, if both *q_u _*and *q_v _*intersect *d′*^−1^(1) \ {*u, v*} (see Figure 3(d)), then we have *xy *∈ *P*. Note further that, if neither *q_u _*nor *q_v _*intersects *d′*^−1^(1) \ {*u, v*} (see Figure 3(b)), then *q_u _*∪ *q_v _*∪ {*uv*} is a path in $G_{i}^{S}$ that does not intersect *d*^−1^(1) and, thus, we have to decrease the number *p *of such paths we are looking for in $G_{j}^{S}$. Formally, let

$$z:=\text{ max}\left\{\begin{array}{c} {\left[d^{\prime },P+uv,p,c-\text{1}\right]}_{j},\\ {\left[d^{\prime },P\cup \left\{uu,vv\right\},p-1,c\right]}_{j},\\ \underset{xx\in P}{\text{max}}\left\{\begin{array}{c} {\left[d^{\prime },\left(P-xx\right)\cup \left\{ux,vv\right\},p,c\right]}_{j},\\ \;{\left[d^{\prime },\left(P-xx\right)\cup \left\{uu,vx\right\},p,c\right]}_{j}, \end{array}\right)\\ \underset{xy\in P}{\text{max}}\left\{{\left[d^{\prime },\left(P-xy\right)\cup \left\{ux,vy\right\},p,c\right]}_{j}.\right) \end{array}\right)$$

**Case 2: ***d*(*u*) = *d*(*v*) = 1. Then, both *u *and *v *are not incident to any edges in $G_{j}^{S}$ and, in $G_{j}^{s_{j}}$, there is just the edge *uv *incident to both. Thus, we set *z *only if *uv *∈ *P*. Formally, let *z *:= [*d′, P *− *uv, p, c*]*_j _*if *uv *∈ *P*.

**Case 3: ***d*(*u*) = 2, *d*(*v*) = 1. Then, there is a path *q *containing *uv *and ending in *v *in $G_{i}^{S}$. If *q *ends in a vertex *x *in *d*^−1^(1) − *v*, we have *vx *∈ *P *and the permutation for *X*(*j*) contains *ux*. Otherwise, we have *vv *∈ *P *and the permutation for *X*(*j*) contains *uu*. Note that, since *v *∈ *d*^−1^(1), we know that *P*(*v*) ≠ ⊥. Formally, for *vx *∈ *P*, let *z *:= [*d′*, (*P *− *vv*) + *uu, p, c*]*_j_*, if *v *= *x *and *z *:= [*d′*, (*P *− *vx*) + *ux, p, c*]*_j_*, otherwise. Finally, let [*d, P, p, c*]*_i _*:= *z *if *uv *∈ *M *and [*d, P, p, c*]*_i _*:= max{*z *+ *ω*(*uv*), [*d, P, p, c*]*_j_*}, otherwise.

**Join: **The join bag *X*(*i*) "glues" the (disjoint) partial solutions of its children together at the vertices of *X*(*i*) = *X*( *j*) = *X*(ℓ). In particular, the degrees in $G_{j}^{S}$ and in $G_{\ell }^{S}$ have to add up to *d*. Furthermore, the permutations *P*_1 _and *P*_2 _for *X*(*j*) and *X*(ℓ), respectively, have to "fit" *P*: For example, let *uv *∈ *P*_1 _and *vw *∈ *P*_2_, implying that there are paths *q_j _*and *q_ℓ _*in $G_{j}^{S}$ and $G_{\ell }^{S}$, respectively, that are connecting *u *and *v *and *v *and *w*, respectively. Then, in $G_{i}^{S}$, there is a single path *q_j _*∪ *q*_ℓ_ connecting *u *and *w *and containing *v *(with *d*(*v*) = 2). Finally, the numbers of paths and cycles have to "fit" *p *and *c*: For example, if the permutations for both *X*(*j*) and *X*(ℓ) contain *uu *(that is, *u *∈ (*P*_1 _∩ *P*_2_)^1^), then $G_{i}^{S}$ contains a path containing *u *that is neither in $G_{j}^{S}$ nor in $G_{\ell }^{S}$. On top of this, the remaining paths must be distributed among $G_{j}^{S}$ and $G_{\ell }^{S}$. Formally, let

$${\left[d,P,p,c\right]}_{i}:=\begin{array}{cccc} \underset{\begin{array}{c} d_{1},d_{2:}X\left(i\right)\to \left\{0,1,2\right\}\\ \forall v\in X\left(i\right),d_{1}\left(v\right)+d_{2}\left(v\right)=d\left(v\right) \end{array}}{\text{max}} & \underset{\begin{array}{c} P_{1},P_{2}\\ P=P_{1}⊔P_{2} \end{array}}{\text{max}} & \underset{\begin{array}{c} p_{1},p_{2},c_{1},c_{2}\\ p_{1}+p_{2}+|{\left(P_{1}\cap P_{2}\right)}^{1}|=p\\ \;c_{1}+c_{2}+|{\left(P_{1}\cap P_{2}\right)}^{2}|=c \end{array}}{\text{max}} & \left\{\begin{array}{c} {\left[d_{1},P_{1},p_{1},c_{1}\right]}_{j}\\ +{\left[d_{2},P_{2},p_{2},c_{2}\right]}_{\ell } \end{array}\right\} \end{array}$$

**Lemma 2 ***The described algorithm is correct, that is, the computed value *[*d, P, p, c*]*_i _corresponds to the semantics*.

**Theorem 1 **SCAFFOLDING* can be solved in O*(tw^tw ^·*σ*_p _· *σ*_c _· *n*) *time, given a width-*tw *tree decomposition of the input instance*.

## Kernel for restricted scaffolding

Towards developing effective preprocessing for SCAFFOLDING, we consider a more restricted problem variant, where all paths and cycles of the solution have to be of certain, respective lengths. NP-hardness of this variant can be inferred with the same reduction as used for SCAFFOLDING[15].

RESTRICTED SCAFFOLDING (RSCA)

**Input: ***G *with perfect matching *M, ω *: *E *→ ℕ with *ω*(*M*) = 0, *σ*_p _∈ ℕ, *σ*_c _∈ ℕ, ℓ_p _∈ ℕ^∗^, ℓ_c _∈ ℕ^∗^, *k *∈ ℕ

**Question: **∃*_X⊆E\M _*s.t. *G *− *X *is a collection of *σ*_p _alternating paths, each of length ℓ_p_,b and *σ*_c _alternating cycles, each of length ℓ_c_, and *ω*(*E*) − *ω*(*X*) ≥ *k*?

The length *l_p _*denotes the number of edges in the paths. It is necessarily odd in a solution of RESTRICTED SCAFFOLDING. In the following, we show that RESTRICTED SCAFFOLDING admits a linear-size problem kernel with respect to the parameter FES (feedback edge set), which is the size of a smallest set of edges whose deletion leaves an acyclic graph. To this end, we present a number of intuitive polynomial-time executable reduction rules that shrink the input graph. The first two rules shrink treelike structures of the instance while the remaining rules contract long chains.

For a *u*-*v*-path *p*, we call *u *and *v *its *outer vertices *while all other vertices of *p *are *inner vertices*. Let *V^○ ^*be the set of vertices that are in some cycle in *G *and let *G^○ ^*= *G*[*V^○^*]. Let *G*^∗ ^= *G*[*V*^∗^] denote the convex hull of *G^○^*, that is, *V*^∗ ^is the set of vertices on some shortest path between some vertices *u, v *∈ *V^○^*. For each *v *∈ *V*^∗^, the tree rooted at *v *that is incident with *v *in *G *− *E*^∗ ^is called the "pendant tree" *T_v_* of *v*.

### Reducing pendant trees

First, we remove isolated paths by cutting them into pieces of length ℓ_p_. Since the correctness of this is trivial, we omit the proof.

**Figure 4:**
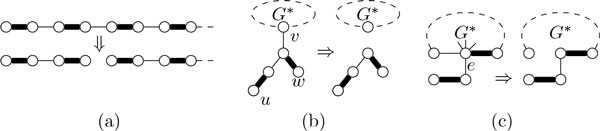
**Illustrations for the tree reduction rules**.

**Tree Rule 1 **(see Figure 4(a)) *Let p be an isolated path in G. If *|*p*| ≥ ℓ_p_*, then split o*ff *a length-*ℓ_p _*path q and decrease *(*σ*_p_, *k*) *by *(1, *ω*(*q*))*. Otherwise, return "NO"*.

The next rule cuts branching edges in pendant trees. To this end, it finds an occurrence of a path of length ℓ_p _that is furthest from the root of the pendant tree.

**Tree Rule 2 **(see Figure 4(b)) *Let T_v _be the pendant tree of some v *∈ *V*^∗^*, let u be a leaf of maximal distance to v in T_v_. Let W be the set of vertices × of T_v _such that a length-*ℓ_p_*alternating u-x-path exists and let w be a vertex in W maximizing *dist*_Tv _*(*v, w*)*. Then, delete from G all edges that are incident with the least common ancestor of u and w but are not on the unique u-w-path in T_v_*.

**Lemma 3 ***Let I *= (*G, M, ω, σ*_p_, *σ*_c_, ℓ_p_, ℓ_c_, *k*) *be a yes-instance of *RESTRICTED SCAFFOLDING* such that G is reduced with respect to Tree Rule 2 and let v *∈ *V*^∗^*. Then, T_v _is a path p that is alternating with respect to M *∩ *E*(*T_v_*) *and *|*p*| < ℓ_p _*and v is an endpoint of p*.

In the following, we assume that *G *is reduced with respect to Tree Rule 2 and, thus, we can reject all instances for which Lemma 3 does not hold. Hence, in the following, we assume that Lemma 3 holds for the input instance. The next reduction rule helps unify the way in which pendant trees (which are now paths) attach to *G*^∗^, simplifying the rest of the presentation.

**Tree Rule 3 **(see Figure 4(c)) *Let T_v _be the pendant tree of some v *∈ *V*^∗ ^*that is incident with an edge e *∈ *E*(*T_v_*) \ *M. Then, delete from G all edges incident with v that are not in M *+ *e*.

### Reducing long paths

In the following, we assume that *G *is reduced with respect to the tree reduction rules presented in the previous section. For *i *∈ ℕ, let *V_i _*denote the set of vertices of *G *that have degree *i *in *G*. The goal in this subsection is to reduce the length of chains of degree-two vertices in *G*. Thus, we consider paths whose inner vertices are all in *V*_2_. We call these paths *deg-2 path*. If the solution has a cycle running through this path, then we cannot modify its length. Therefore, we focus on paths that are guaranteed to not be in a cycle in the solution:

The path reduction rules are based on the idea that the path segments that a deg-2 path *p *in *G *is split into by the solution are recurring, that is, if the solution contains the third edge of *p*, then it also contains the 3 + (ℓ_p _+ 1)^th ^edge of *p*. This gets slightly more complicated if there are pendants in *p*, but first, let us consider paths without any pendants.

**Figure 5:**
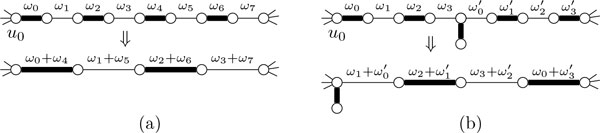
**Illustrations for the first path reduction rules**.

**Path Rule 1 **(see Figure 5(a)) *Let p *= (*u*_0_, *u*_1_, . . .) *be a deg-2 path with *|*p*| > max{ℓ_p_, ℓ_c_} + ℓ_p _+ 1 *and let e_i _*:= *u_i_u_i+1 _for all i. Then, for all e_i _with i *≤ ℓ_p_*, add ω*(*e_i_*) *to ω*(*e*_ℓp +*i*+1_) *and contract e_i_. Finally, decrease σ*_p _*by 1*.

The remaining two rules deal with deg-2 paths containing pendants. Note that any path in the solution that contains the pendant can only "continue" in two directions.

**Path Rule 2 **(see Figure 5(b)) *Let p *= (*u*_0_, *u*_1_, . . ., *u*_ℓp +1 _= *w*) *and q *= (*w *= *v*_0_, *v*_1_, . . ., *v*_ℓp +1_) *be deg-2 paths and let T_w _contain *γ > 0 *edges. For all i *≤ ℓ_p_*, let e_i _*:= *u_i_u_i+1_, let f_i _*:= *v_i_v_i+1_ and let i*^+ ^:= (*i *− γ) mod (ℓ_p_ + 1)*. Then, for all i *≤ ℓ_p_*, add ω*(*e_i_*) *to ω*( *f_i+_*) *and contract e_i_. Finally, decrease σ*_p _*by 1*.

In case of two pendants, the solution is restricted by the distance between the pendants. If we can infer what the solution does by this length, we implement this right away, otherwise, we can represent the choices that a solution can take with a single pendant.

**Figure 6:**
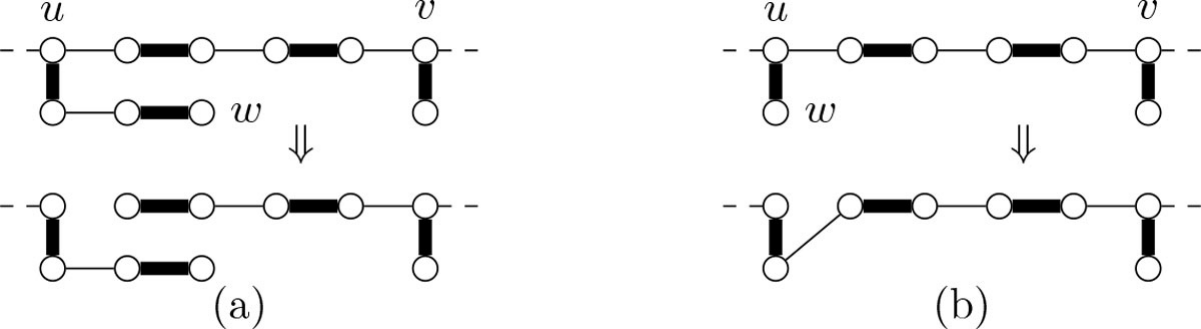
**Illustrations for Path Rule 3**.

**Path Rule 3 **(see Figure 6) *Let p *= (*x*_0_, *x*_1_, . . .) *be a u-v-deg-2 path such that u, v *∈ *V*^∗^*. Let *γ*_u _*> 0 *and *γ*_v _*> 0 *be the number of edges in T_u _and T_v_, respectively, and let w be the vertex at maximum distance to u in T_u_. Let *γ := |*p*| mod (ℓ_p_ + 1) *and let G′ be the result of replacing ux*_1 _*by wx*_1 _*of the same weight in G*.

(*1*) *If *γ + γ*_u_*≠ f ℓ_p_ + 1 = γ + γ*_v_, then delete ux*_1 _(*see *Figure 6(a)).

(*2*) *If *γ + γ*_u _*= ℓ_p_ + 1 ≠ f γ + γ*_v_, then delete vx*_|*p*|−1_ .

(*3*) *If *γ + γ*_u _*= ℓ_p_ + 1 = γ + γ*_v_, then return G′ *(*see *Figure 6(b)).

(*4*) *If *γ = 1 *and *γ*_u _*+ γ*_v _*+ 1 = ℓ_p_*, then return G′ *(*see *Figure 6(b)).

(*5*) *If *γ = 1 *and *γ*_u _*+ γ*_v _*+ 1 ≠ ℓ_p_*, then delete ux*_1 _(*see *Figure 6(a)).

(*6*) *If *γ ≠ 1 *and *γ + γ*_u _*+ γ*_v _*≡ ℓ_p _mod (ℓ_p_ + 1)*, then delete all edges e *∈ *E*(*G*) \ (*M *∪ {*ux*_1_}).

(*7*) *In all other cases, return "NO"*.

Finally, we can show that an input graph that is reduced with respect to these rules cannot be larger than 11ℓ_p_ · FES(*G*) or 11ℓ_c_ · FES(*G*). To prove this, let *G*^† ^= (*V*^†^, *E*^†^) be the result of contracting all degree-2 vertices in *G*^∗^.

**Lemma 4 ***Let G be reduced with respect to all presented reduction rules. Then*, |*V*| ≤ ℓ · (|*V*^†^| + 3|*E*^†^|) *with *ℓ := max{ℓ_c_, ℓ_p_}.

*Proof *By Lemma 3, we know that ∀*_v∈V_*†|*E*(*T_v_*)| < ℓ_p_. Therefore, if Lemma 4 is false, there is an edge *uv *∈ *E*^† ^such that there are > 3ℓ vertices between *u *and *v *(i.e. *uv *is a contraction of more than 3ℓ edges of *G*). Nevertheless, by irreducibility with respect to Path Rule 3, there is at most one vertex *w *between *u *and *v *such that *T_w_*is not empty and the distance between *u *and *v *cannot be greater than 2ℓ + 1 (by irreducibility with respect to Path Rule 1 and 2). So, |*E*(*T_w_*)| ≥ ℓ, contradicting Lemma 3.

**Theorem 2 **RESTRICTED SCAFFOLDING* admits a kernel containing at most *11ℓ · FES(*G*) *vertices and *(11ℓ + 1) · FES(*G*) *edges where *ℓ := max{ℓ_p_, ℓ_c_}.

## Results

### Data

In our experiments, we worked with two datasets, all derived from real genomes (see Table 1). The first one is a set of eleven contig sets, produced from real genomes using the following process: first, the genomes where taken off the NCBI *nucleotide *database (http://www.ncbi.nlm.nih.gov). Then, for each of them, a set of simulated paired-end reads was generated with the tool wgsim ([28]), with default parameters and a 20X mean covering depth. Thereafter, assembly was performed with the tool minia ([29]) with a *k*-mer size *k *= 29. Reads were mapped on the contigs with bwa ([30]), with default parameters and using the sampe method. The second dataset is composed of five scaffold graphs, already presented in [31] and [17]. Some of them have been produced by simulating reads, other come from real paired-end reads libraries (see Table 1). Finally, using the scaftools previously developed in [15,31], we produced the scaffold graphs corresponding to these datasets. See [31] for a more detailed explanation of this pipeline.

**Table 1:** Details on the second dataset.

Genome	Reads library	Assembly tool	Mapping tool
staphylo	short jump library (from GAGE [32])	velvet [33]	bwa [30]

ecoli	Illumina reads library SRR001665	velvet	bowtie [34]

ypco92	simulated with wgsim	minia [35]	bwa

wolbachia	simulated with toyseq for the Variathon experiment [36]	minia	bwa

arabido	Illumina reads library SRR616966	velvet	bowtie

The aim of this process is first to produce a benchmark of test graphs that are more realistic than uniformly generated graphs to test our algorithms on, second to study the different parameters that may be interesting to consider for parameterized algorithms, and finally to help to design a more realistic and convenient scaffold graph generator allowing to generate graphs directly, avoiding the complicated pipeline described above (see Table 2 and Table 3). The second dataset was used to study the influence of a preprocessing operation on the scaffold graph, aiming at filtering low informative edges. Simplistically, we removed respectively edges with weight less than 3, 6 and 10. Results are presented in Table 3. We already know that this operation improves the quality of the produced scaffolds, when compared to the original reference genome. Here we would like to observe the behavior of our putative interesting structural parameters according to this filtering.

**Table 2:** Scaffold graphs parameters.

Data	|*V*|	|*E*|	Min/Max/Avg degree	FES	FVS (ub)	tw(ub)	*h*	*dcy*
anopheles^Ch^	84090	113497	1 / 51 / 2.70	29851	17962		12	3

anthrax^Ch^	8110	11013	1 / 7 / 2.72	2906	1232	574	7	2

ebola^Co^	34	43	1 / 5 / 2.53	10	6	3	4	2

gloeobacter^Ch^	9034	12402	1 / 12 / 2.75	3375	2484	639	8	3

lactobacillus^Ch^	3796	5233	1 / 12 / 2.76	1439	804	260	8	2

monarch^Mt^	28	33	1 / 4 / 2.36	6	4	3	4	2

pandora^Co^	4902	6722	1 / 7 / 2.74	1822	1277	327	7	2

pseudomonas^Ch^	10496	14334	1 / 9 / 2.73	3851	2692	752	8	2

rice^Cp^	168	223	1 / 6 / 2.65	56	31	9	5	2

sacchr3^Ch^	592	823	1 / 7 / 2.78	232	142	43	6	2

sacchr12^Ch^	1778	2411	1 / 10 / 2.12	637	575	124	7	2

FES = feedback edge set, FVS (ub) = upper bound on the feedback vertex set, *dcy *= degeneracy. Superscripts: Ch = Chromosome, Cp = Chloroplast, Co = Complete, Mt = Mitochondrion

**Table 3:** Scaffold graph parameters for select genomes and different cut-off thresholds: 0, 3, 6, and 10.

Data & threshold (|*V*|)		|*E*|	Min/Max/Avg degree	FES	FVS(ub)	tw(ub)	*h*	*dcy*
arabido (345232)	0 3 6 10	318984 252762 230333 215094	1 / 31 / 1.85 1 / 14 / 1.46 1 / 9 / 1.33 1 / 9 / 1.25	43593 8024 3247 1224	15703 5881 2814 1020	10610 792 74 51	18 9 8 8	4 3 2 2

ecoli (1732)	0 3 6 10	8142 4043 3105 2695	2 / 46 / 9.40 2 / 23 / 4.67 2 / 18 / 3.59 2 / 16 / 3.11	6411 2312 1374 964	644 406 327 278	551 303 162 102	32 16 13 11	9 4 4 3

staphylo (602)	0 3 6 10	4765 1743 1017 790	1 / 128 / 15.83 1 / 58 / 5.79 1 / 22 / 3.37 1 / 16 / 2.62	4164 1152 464 279	167 113 78 65	124 57 25 14	52 25 14 11	28 11 5 4

wolbachia (560)	0 3 6 10	1036 523 459 399	1 / 56 / 3.70 1 / 15 / 1.86 1 / 15 / 1.63 1 / 5 / 1.43	481 47 19 12	106 30 16 11	26 3 2 2	11 6 5 5	3 2 2 2

ypco92 (2656)	0 3 6 10	3465 2849 2651 2525	1 / 8 / 2.61 1 / 6 / 2.15 1 / 5 / 1.99 1 / 5 / 1.90	821 241 99 73	313 136 86 68	191 38 3 2	7 6 5 5	2 2 2 2

FES = feedback edge set, FVS (ub) = upper bound on the feedback vertex set, tw(ub) = upper bound on the treewidth (by greedy fill-in), *h *= h-index, *dcy *= degeneracy.

### Graph parameters

Table 2 presents some parameters of the generated graphs. The h-index is the maximum number such that the graph contains *h *vertices of degree at least *h*. It measures the degree of connectivity of a graph. The feedback edge set (FES) is the size of a smallest set of edges whose deletion leaves an acyclic graph. The degeneracy is the smallest value *d *for which every subgraph has a vertex of degree at most *d*. It is a kind of measure of sparsity of the graph. We notice that scaffold graphs look quite sparse, with few vertices of high degrees and a feedback edge set number that is usually significantly lower than the number of vertices. While degree-based graph parameters like the degeneracy *d *are tiny in all instances, we recall that our problem generalizes Hamiltonian Cycle, which is already N P-hard on 3-regular graphs. However, Table 2 shows that, for instances that we expect to be seen in practice, these measures can be assumed constant and, thus, it might be worth considering a combination involving these parameters.

Table 3 shows the same statistics for the second set of graphs where edges of small weight (that is, low confidence) were discarded (for weight thresholds of 10, 6, 3 and 0, yielding the original graph with all edges present). We notice that even with a light filtering, the parameters of the scaffold graph are considerably lowered, confirming that raw data suffers from significant noise. Thus, filtering low quality information not only increases the quality of the scaffolding, but also may lead to a significant leap to tractability. Note that, at the time of writing this article, the *Staphylococcus Aureus *genome is no longer available in the NCBI Nucleotide database ("This RefSeq genome was suppressed because updated RefSeq validation criteria identified problems with the assembly or annotation.") and the *Escherichia Coli *genome has been updated since the presented version. Errors in assemblies are quite frequent ([37]) and yield additional issues in scaffolding. Having a better idea of the structure of a classical scaffold graph, and some simple criteria to determine what is anomalous and what is normal (subgraphs induced by repeats for instance) would be of real interest for the analysis of genomes.

### Implementation and first results

We implemented the dynamic programming algorithm presented in Section 11 in C++ using boost and the treewidth optimization library TOL [38]. We ran it on a selection of the generated data sets (see Section 11) for which the greedy fill-in heuristic produced tree decompositions of width at most 45. We chose (*σ*_p_, *σ*_c_) = (3, 1) for the ebola and monarch genomes and (*σ*_p_, *σ*_c_) = (20, 3) for the more complicated inputs. The tests were run on an AMD Opteron(tm) Processor 6376 at 2300 MHz.

**Figure 7:**
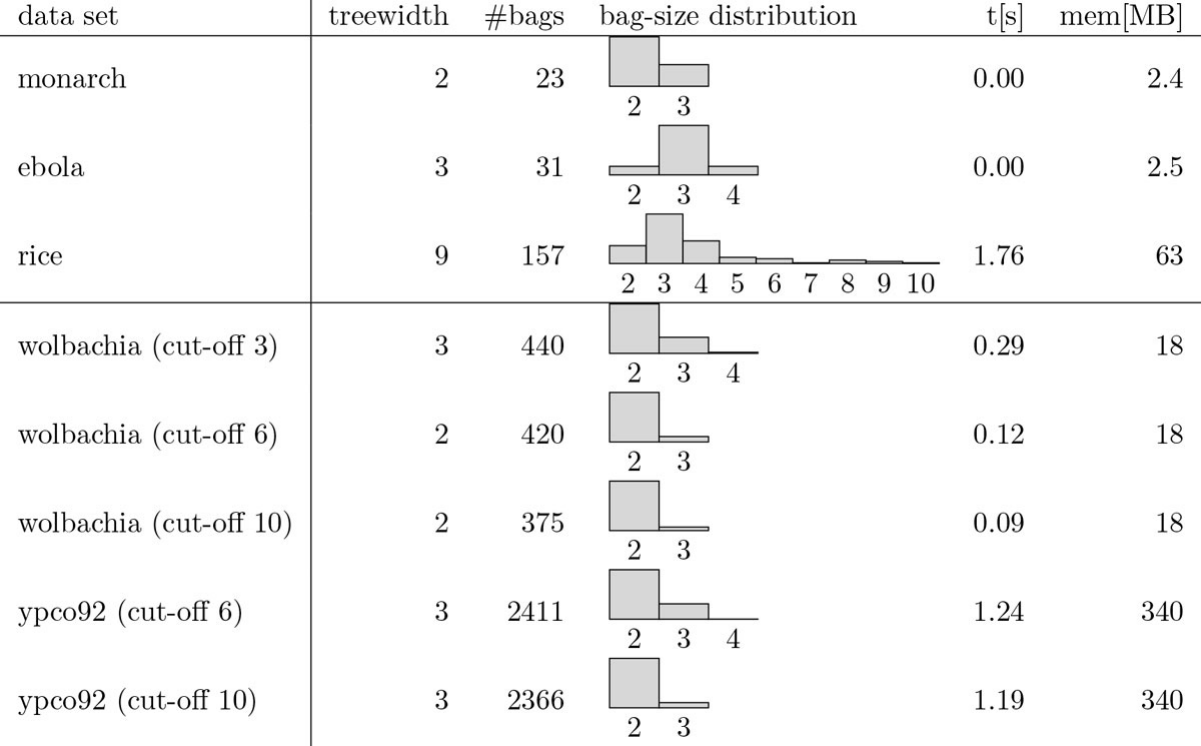
**Information on the tree decomposition and resource requirements of running the algorithm for select instances with small treewidth**. We chose (*σ*_p_, *σ*_c_) = (3, 1) for ebola and monarch, (20, 3) for rice and (256, 16) for the second data set (with edge cut-offs).

**Figure 8:**
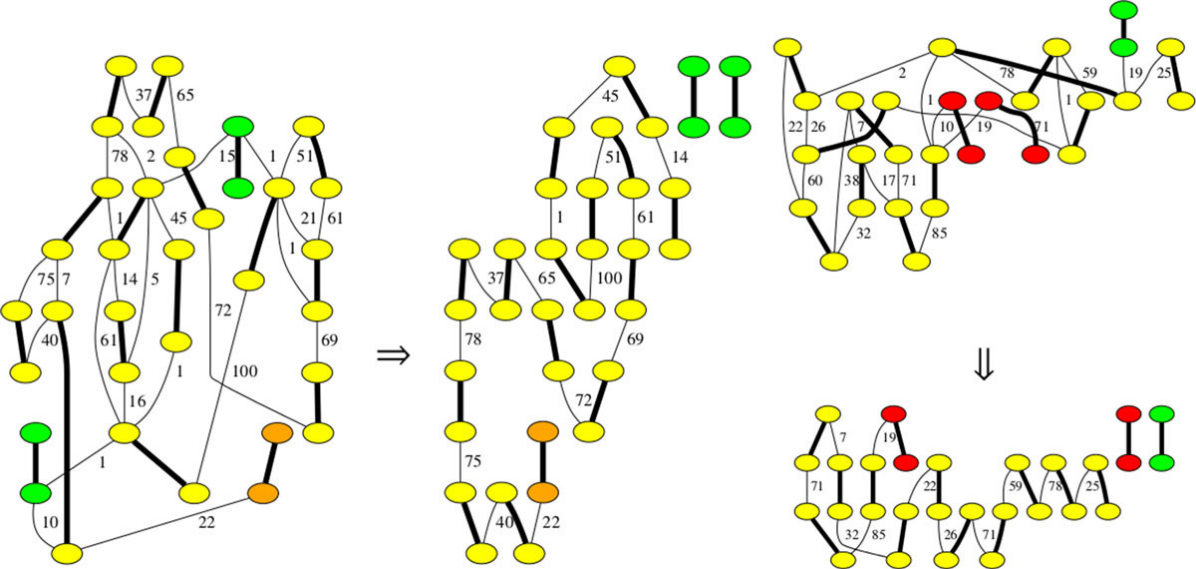
**Optimal solutions for the ebola (left) and monarch (right) graphs**. Edges have their weights on the right. Matching edges are bold and normalized to weight 0.

Figure 7 shows running times and memory consumption needed to produce a solution as well as details regarding the used tree decompositions. Figure 8 shows optimal solutions for the two smallest data-sets: ebola and monarch. To validate the proposed scaffolding, we compared the output to the alignment of the contigs on the reference sequence using megablast ([39]). In the case of the ebola genome, the two isolated contigs (in green) are small (about 150 bp). One of them is placed between the orange contig and its neighbor, the other one finds its place at the other extremity of the chain. In the input scaffold graph, we notice that they are linked to the wrong node, we suppose this is due to their small size, disturbing the alignment step. For the monarch mitochondrion, however, two of the contigs (appearing in red) did not match on the sequence, meaning that the assembly yields some errors. The isolated contig is also small and not correctly connected in the scaffold graph.

**Figure 9:**
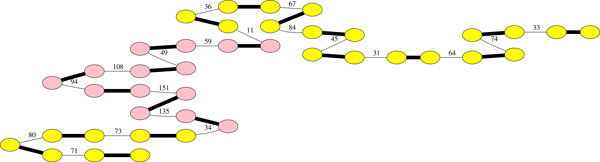
**Scaffold in the rice chloroplast genome, including the inverted repeat**.

Concerning the rice chloroplast, among the 84 contigs, only three were misplaced. All three of them are small (< 130 bp), two of them strongly overlap and the third has two occurrences in the reference genome, one complete and one partial. The seven remaining scaffolds follow exactly the right relative order and orientation of the contigs on the reference genome. Chloroplast genomes have a particularity which make them interesting as data for scaffolding. They present an inverted repeat region of approximately 20 kbp [40]. Figure 9 focuses on one of the scaffolds, where this inverted repeat is shown in pink. The other occurrence is not present in the scaffolding. To notice, the weights inside this repeat are in average higher than outside, which is totally expected since the read cover is approximately doubled for these sequences. Thus, areas of the graph with higher weight would lead to repeat hypothesis, if we are confident in the homogeneity of the cover.

## Discussion and conclusion

In this paper, we considered exact approaches to the NP-hard SCAFFOLDING problem which is an integral part of genome sequencing. We showed that it can be solved in polynomial time on trees or graphs that are close to being trees (constant treewidth) by a dynamic programming algorithm. We proved a linear-size problem kernel for the parameter "feedback edge set" for a restricted version in which the lengths of paths and cycles are fixed. We implemented an exact algorithm solving SCAFFOLDING in *f*(tw) · poly(*n*) time and evaluated our implementation experimentally, supporting the claim that this method produces high-quality scaffolds. Our experiments are run on data sets that are based on specific real-world genomes, which we also examined to identify a number of interesting parameters that may help design parameterized algorithms and random scaffold graph generators that produce more realistic instances. We are currently transferring the preprocessing rules to the general problem variant. We are highly interested in further graph classes that are closer to real-world instances than trees and on which the problem might be polynomial-time solvable. From an algorithmic point of view, we remark that only few bags of the used tree decompositions are small (see Figure 7). Thus, we envision a hybrid strategy of branching on the vertices in the largest bags before running the dynamic-programming algorithm and using this "distance *x *to treewidth-*x*" parameter to re-analyze the problem.

We intend to perform more extensive tests on diverse datasets, in particular comparing the quality of this approach to existing ones using, for instance, the criteria presented in [9]. This work demands reliable benchmarks, with up-to-date and well assembled genomes. From a bioinformatics point of view, we lay the groundwork for a careful analysis of the scaffold graph, as well as a tool to speed up the above algorithms, as a help to analyze the quality of the assembly, and maybe the structure of the genome itself.

## Appendix

*Proof of Lemma 1 *The proof is by induction over the distance of *v *to *r *(descending) and, in case of ties, *j *(ascending).

**Induction Base: **The statement holds for all vertices *v *if *j *= 0 since $T_{0}^{v}=T\left[\left\{v\right\}\right]$ does not contain edges.

**Induction Step (**≥**): **First, we show that [*c, i, j*]*_v _*≥ *ω*(*S*). To this end, let *u *:= *s_v_*[*j*] and, noting that all vertices except maybe *v *of $T_{j}^{v}$ are incident with edges in *M*, let *q *denote the path in *G*[*S *∪ *M*] containing *u*. Let *w *be the vertex paired with *v *by *M*. Let *α *denote the number of edges in *q *∩ *E*(*T_u _*+ *v*) \ *M *incident with *u *and let *β *≤ *c *denote the number of edges in $q\cap E\left(T_{j-1}^{v}\right)\backslash M$ incident with *s_v_*[1..(*j *− 1)]. Let *S_u _*:= U*_p∈S_ p *∩ *E*(*T_u_*) and let $S_{j-1}:=\cup_{p\in S}p\cap E\left(T_{j-1}^{v}\right)$ and note that *S _u _*and *S _j−1 _*are path covers of *T_u _*and $T_{j-1}^{v}$, respectively. Thus, by induction hypothesis,

(1) $$\omega \left(S_{u}\right)\leq {\left[\alpha ,∥S_{u}{∥}_{p},\infty \right]}_{u}\;\text{and}\;\omega \left(S_{j-1}\right)\leq {\left[\beta ,∥S_{j-1}{∥}_{p},j-1\right]}_{v}.$$

**Case 1: ***uv *∈ *M *and *c *= 0. Then, $∥S{∥}_{p}=∥S_{u}{∥}_{p}^{T_{u}+v}+∥S_{j-1}{∥}_{p}=∥S_{u}{∥}_{p}+\left(1-\alpha \right)+∥S_{j-1}{∥}_{p}$ and, thus,

$${\left[c,i,j\right]}_{v}\overset{line\;6}{\geq }{\left[\alpha ,∥S_{u}{∥}_{p},\infty \right]}_{u}+{\left[0,i-\left(∥S_{u}{∥}_{p}-\alpha +1\right),j-1\right]}_{v}\overset{\left(1\right)}{\geq }\omega \left(S_{u}\right)+\omega \left(S_{j-1}\right)=\omega \left(S\right).$$

**Case 2: ***uv *∈ *M *and *c *= 1. Then, *β *= 1. Furthermore, the path containing *u *is split over *S _u _*+ *uv *and *S _j-1_*, implying $∥S{∥}_{p}=∥S_{u}{∥}_{p}^{T_{u}+v}+∥S_{j-1}{∥}_{p}-1=∥S_{u}{∥}_{p}-\alpha +∥S_{j-1}{∥}_{p}$. Thus,

$${\left[c,i,j\right]}_{v}\overset{line\;7}{\geq }{\left[\alpha ,∥S_{u}{∥}_{p},\infty \right]}_{u}+{\left[0,i-\left(∥S_{u}{∥}_{p}-\alpha \right),j-1\right]}_{v}\overset{\left(1\right)}{\geq }\omega \left(S_{u}\right)+\omega \left(S_{j-1}\right)=\omega \left(S\right).$$

**Case 3: ***uv *∉ *M *and *c *= 0. Then, ||*S*||*_p _*= ||*S_u_*||*_p_*+ ||*S _j−1_*||*_p _*and we have

$${\left[c,i,j\right]}_{v}\overset{line9}{\geq }{\left[\alpha ,∥S_{u}{∥}_{p},\infty \right]}_{u}+{\left[0,i-∥S_{u}{∥}_{p},j-1\right]}_{v}\overset{\left(1\right)}{\geq }\omega \left(S_{u}\right)+\omega \left(S_{j-1}\right)=\omega \left(S\right).$$

**Case 4: ***uv *∉ *M *and *c *= 1. If *uv *∉ *S *, then *β *= 1. Furthermore, ||*S*||*_p _*= ||*S_u_*||*_p _*+ ||*S_j−1_*||*_p_* and we have

$${\left[c,i,j\right]}_{v}\overset{line10}{\geq }{\left[\alpha ,∥S_{u}{∥}_{p},\infty \right]}_{u}+{\left[1,i-∥S_{u}{∥}_{p},j-1\right]}_{v}\overset{\left(1\right)}{\geq }\omega \left(S_{u}\right)+\omega \left(S_{j-1}\right)=\omega \left(S\right).$$

Otherwise, *uv *∈ *S *, implying *α *= *β *= 0. If *w *∉ *s_v_*[1..*j*], then $∥S{∥}_{p}=∥S_{u}+uv{∥}_{p}^{T_{u}+v}+∥S_{j-1}{∥}_{p}=∥S_{u}{∥}_{p}+∥S_{j-1}{∥}_{p}$ and

$${\left[c,i,j\right]}_{v}\overset{line10}{\geq }\omega \left(uv\right)+{\left[0,∥S_{u}{∥}_{p},\infty \right]}_{u}+{\left[0,i-∥S_{u}{∥}_{p},j-1\right]}_{v}\overset{\left(1\right)}{\geq }\omega \left(uv\right)+\omega \left(S_{u}\right)+\omega \left(S_{j-1}\right)=\omega \left(S\right).$$

Otherwise, *w *∈ *s_v_*[1..*j*] and the path containing *u *is split over *S _u _*and *S _j−1_*. Thus, $∥S{∥}_{p}=∥S_{u}+uv{∥}_{p}^{T_{u}+v}+∥S_{j-1}{∥}_{p}-1=∥S_{u}{∥}_{p}+∥S_{j-1}{∥}_{p}-1$, implying

$${\left[c,i,j\right]}_{v}\overset{line10}{\geq }\omega \left(uv\right)+{\left[0,∥S_{u}{∥}_{p},\infty \right]}_{u}+{\left[0,i-\left(∥S_{u}{∥}_{p}-1\right),j-1\right]}_{v}\overset{\left(1\right)}{\geq }\omega \left(uv\right)+\omega \left(S_{u}\right)+\omega \left(S_{j-1}\right)=\omega \left(S\right).$$

**Induction Step (**≤**): **Next, we show that [*c, i, j*]*_v _*≤ *ω*(*S *) by proving that a *i*-path cover *S′ *for $T_{j}^{v}$ of weight [*c, i, j*]*_v _*exists and, thus, [*c, i, j*]*_v _*= *ω*(*S′*) ≤ *ω*(*S *) by optimality of *S*. To this end, let *u *:= *s_v_*[*j*] and let *w *:= *M*(*v*).

**Case 1: ***w *= *u *and *c *= 0. By line 6, there are ℓ and *α *such that [0, *i, j*]*_v_*= [*α*, ℓ, ∞]*_u _*+[0, *i*− (ℓ − *α *+ 1), *j *− 1]*_v_*. By induction hypothesis, there are path covers *S _u _*and *S _j−1 _*corresponding to [*α*, ℓ, ∞]*_u _*and [0, *i *− (ℓ − *α *+ 1), *j *− 1]*_v_*, respectively. Then, $S^{\prime }:=S_{u}⊎S_{j-1}$ is a path cover for $T_{j}^{v}$ and $∥S^{\prime }{∥}_{p}=∥S_{u}{∥}_{p}^{T_{u}+v}+∥S_{j-1}{∥}_{p}=∥S_{u}{∥}_{p}+1-\alpha +∥S_{j-1}{∥}_{p}=i$. Furthermore,

$$\omega \left(S^{\prime }\right)=\omega \left(S_{u}\right)+\omega \left(S_{j-1}\right)\overset{ind.\;hyp.}{=}{\left[\alpha ,\ell ,\infty \right]}_{u}+{\left[0,i-\left(\ell -\alpha +1\right),j-1\right]}_{v}\overset{line\;6}{=}{\left[0,i,j\right]}_{v}.$$

**Case 2: ***u *= *w *and *c *= 1. By line 7, there are ℓ and *α *such that [1, *i, j*]*_v_*= [*α*, ℓ, ∞]*_u_*+[1, *i*− (ℓ − *α*), *j *− 1]*_v_*. By induction hypothesis, there are path covers *S _u _*and *S _j−1_*corresponding to [*α*, ℓ, ∞]*_u _*and [1, *i *− (ℓ − *α*), *j *− 1]*_v_*, respectively. Then, *S′ *:= *S _u_*∪ *S _j−1 _*is a path cover for $T_{j}^{v}$ and $∥S^{\prime }{∥}_{p}=∥S_{u}{∥}_{p}^{T_{{}^{u}}+v}+∥S_{j-1}{∥}_{p}-1$ since *S _j−1_*contains an edge incident to *v*. Thus, $∥S^{\prime }{∥}_{p}=∥S_{u}{∥}_{p}+∥S_{j-1}{∥}_{p}-\alpha =i$. Furthermore,

$$\omega \left(S^{\prime }\right)=\omega \left(S_{u}\right)+\omega \left(S_{j-1}\right)\overset{ind.hyp.}{=}{\left[\alpha ,\ell ,\infty \right]}_{u}+{\left[1,i-\left(\ell -\alpha \right),j-1\right]}_{v}\overset{line7}{=}{\left[1,i,j\right]}_{v}.$$

**Case 3: ***w ≠ u *and *c *= 0. By line 9, there are ℓ and *α *such that [0, *i, j*]*_v _*= [*α*, ℓ, ∞]*_u _*+ [0, *i *− ℓ, *j *− 1]*_v_*. By induction hypothesis, there are path covers *S _u _*and *S _j−1 _*corresponding to [*α*, ℓ, ∞]*_u _*and [0, *i *− ℓ, *j *− 1]*_v_*, respectively. Since *c *= 0 we have *uv *∉ *S _u _*and, thus, $S^{\prime }:=S_{u}⊎S_{j-1}$ is a path cover for $T_{j}^{v}$ and $∥S^{\prime }{∥}_{p}=∥S_{u}{∥}_{p}+∥S_{j-1}{∥}_{p}=i$. Furthermore,

$$\omega \left(S^{\prime }\right)=\omega \left(S_{u}\right)+\omega \left(S_{j-1}\right)\overset{ind.\;hyp.}{=}{\left[\alpha ,\ell ,\infty \right]}_{u}+{\left[0,i-\ell ,j-1\right]}_{v}\overset{line9}{=}{\left[0,i,j\right]}_{v}.$$

**Case 4: ***w ≠ u *and *c *= 1.

**Case 4a: **There are ℓ and *α *such that [1, *i, j*]*_v _*= [*α*, ℓ, ∞]*_u _*+ [1, *i *− ℓ, *j *− 1]*_v _*(see line 10). By induction hypothesis, there are path covers *S _u _*and *S _j−1 _*corresponding to [*α*, ℓ, ∞]*_u _*and [1, *i *− ℓ + 1, *j *− 1]*_v_*, respectively. Since *uv *∉ *M*, some edge incident to *u *in *T_u _*is in *M*. Then, $S^{\prime }:=S_{u}⊎S_{j-1}$ is a path cover for $T_{j}^{v}$ and ||*S′*||*_p _*= ||*S _u_*||*_p_*+ ||*S _j−1_*||*_p_*= *i*. Furthermore,

$$\omega \left(S^{\prime }\right)=\omega \left(S_{u}\right)+\omega \left(S_{j-1}\right)\overset{ind.\;hyp.}{=}{\left[\alpha ,\;\ell ,\;\infty \right]}_{u}+{\left[1,\;i-\ell ,j-1\right]}_{v}\overset{line10}{=}{\left[1,\;i,\;j\right]}_{v}.$$

**Case 4b: **There is some ℓ such that [1, *i, j*]*_v _*= *ω*(*uv*) + [0, ℓ, ∞]*_u _*+ [1, *i *− (ℓ − 1), *j *− 1]*_v _*(see line 10). Then, *w *∈ *s_v_*[1..*j*] and, by induction hypothesis, there are path covers *S _u _*and *S _j−1_* corresponding to [0, ℓ, ∞]*_u _*and [1, *i*−ℓ+1, *j*−1]*_v_*, respectively. Then, *S′ *:= (*S _u_*+*uv*) ∪ *S _j−1_*is a path cover for $T_{j}^{v}$ and *w*,*v *and *u *are on the same path *p *in $T_{j}^{v}\left[S^{\prime }\cup M\right]$. Since *u *is incident to an edge of *M *in *T_u_*, we know that *p *does not end in *u*. Thus, ||*S′*||*_p _*= ||*S _u_*||*_p_*+ ||*S _j−1_*||*_p_*− 1 = *i*. Furthermore,

$$\omega \left(S^{\prime }\right)=\omega \left(S_{u}\right)+\omega \left(S_{j-1}\right)+\omega \left(uv\right)\overset{ind.\;hyp.}{=}{\left[0,\;\ell ,\;\infty \right]}_{u}+{\left[1,\;i-\left(\ell +1\right),j-1\right]}_{v}\overset{line10}{=}{\left[1,\;i,\;j\right]}_{v}.$$

**Case 4c: **There is some ℓ such that [1, *i, j*]*_v _*= *ω*(*uv*) + [0, ℓ, ∞]*_u _*+ [1, *i *− ℓ, *j *− 1]*_v _*(see line 10). Then, *w *∉ *s_v_*[1..*j*] and, by induction hypothesis, there are path covers *S _u _*and *S _j−1 _*corresponding to [0, ℓ, ∞]*_u _*and [1, *i *− ℓ, *j *− 1]*_v_*, respectively. Thus, *S′ *:= (*S _u _*+ *uv*) ∪ *S _j−1 _*is a path cover for $T_{j}^{v}$. Since *u *is incident to an edge of *M *in *T_u_*, we have ||*S′*||*_p _*= ||*S _u_*||*_p _*+ ||*S _j−1_*||*_p _*= *i*. Furthermore,

$$\omega \left(S^{\prime }\right)=\omega \left(S_{u}\right)+\omega \left(S_{j-1}\right)+\omega \left(uv\right)\overset{ind.hyp.}{=}{\left[0,\ell ,\infty \right]}_{u}+{\left[1,i-\ell ,j-1\right]}_{v}\overset{line10}{=}{\left[1,i,j\right]}_{v}.$$

Proof of Section 11

*Proof of Lemma 2 *The proof is by induction on the distance of *i *to *r *(descending). In the induction base, *i *is a leaf of *T *and *X*(*i*) = ∅ and *G_i _*is empty. Thus, the domains of *d *and *P *are empty. Thus, [∅, ∅, 0, 0]*_i _*= 0 and all other entries are −∞.

For the induction step, we distinguish the possible bag types of *X*(*i*) with children *X*( *j*) and *X*(ℓ) (possibly *j *= ℓ):

**Introduce vertex ***v***: **Since *G_i _*does not contain edges incident to *v*, only tuples with *d*(*v*) = 0 and *P*(*v*) = ⊥ are valid.

**Forget vertex ***v***: **Let *S_i_* be a maximum weight set that is eligible for (*d, P, p, c, i*). We show that [*d, P, p, c*]*_i _*= *ω*(*S_i_*).

"≤":

**Case 1: **[*d, P, p, c*]*_i _*= [*d*[*v *→ 1], *P *+ *vv, p *− 1, *c*] *_j_*. By induction hypothesis, there is a set *S_j _*corresponding[1] to [*d*[*v *→ 1], *P *+ *vv, p *− 1, *c*] *_j_*. We show that *S_j _*is eligible for (*d, P, p, c, i*) and, thus, *ω*(*S _i_*) ≥ *ω*(*S _j_*) = [*d, P, p, c*]*_i_*. Since *X*(*i*) ⊂ *X*( *j*) and all paths between vertices in *d*^−1^(1) that are represented by *P *+ *vv *are also represented by *P*, the first two conditions are satisfied by *S_j _*for *i*. Since ${\text{deg}}_{G_{j}^{S_{j}}}\left(v\right)=1$, there is a path *q *in $G_{j}^{s_{j}}$ containing *v*. But since *v *∉ *d*^−1^(1), we know that $G_{i}^{S_{i}}$ contains one path more that does not intersect *d*^−1^(1). Thus, $G_{i}^{S_{i}}$ contains *p *paths that do not intersect *d*^−1^(1) and *S_j _*satisfies the third condition.

**Case 2: **[*d, P, p, c*]*_i _*= [*d*[*v *→ 1], (*P *− *uu*) + *uv, p, c*] *_j _*for some *uu *∈ *P*. By induction hypothesis, there is a set *S_j _*corresponding to [*d*[*v *→ 1], (*P*−*uu*)+*uv, p, c*] *_j_*. Since *uv *∈ (*P *− *uu*) + *uv*, there is a *u*-*v*-path *q *in $G_{j}^{s_{j}}$ (by Definition 2(2)) and *q *intersects *d*^−1^(1) in *u*. Thus, $G_{i}^{S_{i}}$ contains *p *paths that do not intersect *d*^−1^(1) and, thus, *S_j _*is eligible for (*d, P, p, c, i*).

**Case 3: **[*d, P, p, c*]*_i _*= [*d*[*v *→ *x*], *P, p, c*] *_j _*for some *x *∈ {0, 2}. By induction hypothesis, there is a set *S_j _*corresponding to [*d*[*v *→ *x*], *P, p, c*] *_j_*. Then, *S_j _*is also eligible for (*d, P, p, c, i*).

"≥": Let $x:={\text{deg}}_{G_{i}^{s_{i}}}\left(v\right)$.

**Case 1: ***x *∈ {0, 2}. Then, *S_i_* is eligible for (*d*[*v *→ *x*], *P, p, c, j*) and, by induction hypothesis, *ω*(*S _i_*) ≤ [*d*[*v *→ *x*], *P, p, c*] *_j _*≤ [*d, P, p, c*]*_i_*.

**Case 2: ***x *= 1. Then, by Definition 2(1), there is a path *q *in $G_{i}^{S_{i}}$ ending in *v*. If *q *has another end *u *in *d*^−1^(1), then *S_i _*is eligible for (*d*[*v *→ 1], (*P *− *uu*) + *uv, p *− 1, *c, j*) and, thus, *ω*(*S _i_*) ≤ [*d*[*v *→ 1], (*P *− *uu*) + *uv, p *− 1, *c*]. Otherwise, *S_i_* is eligible for (*d*[*v *→ 1], *P *+ *vv, p *− 1, *c, j*). In both cases, *ω*(*S _i_*) ≤ [*d, P, p, c*]*_i_*.

**Introduce edge ***uv***: **Let *S _i_*correspond to [*d, P, p, c*]*_i_*. We show that [*d, P, p, c*]*_i_*= *ω*(*S _i_*). Let *d′ *and *z *be as described in the dynamic programming and note that $G_{j}^{=}G_{i}^{-}uv.$ "≤": First, if [*d, P, p, c*]*_i _*= [*d, P, p, c*] *_j_*, then, by induction hypothesis, there is a set *S _j_*corresponding to [*d, P, p, c*] *_j _*and *uv *∉ *M*. Then, $G_{j}^{s_{j}}=G_{i}^{s_{j}}$ implying that *S_j _*is eligible for (*d, P, p, c, i*) and, thus, *ω*(*S _i_*) ≥ *ω*(*S _j_*) = [*d, P, p, c*]*_i_*.

In the following, we proceed in a similar manner for the case that [*d, P, p, c*]*_i _*= *z *+ *ω*(*uv*): To show [*d, P, p, c*]*_i _*≤ *ω*(*S _i_*), we consider a set *S_j _*that corresponds to the entry for *X*( *j*) from which [*d, P, p, c*]*_i _*is computed and whose existence is granted by induction hypothesis. Then, we show that *S _j_*+ *uv *is eligible for (*d, P, p, c, i*), implying *ω*(*S _i_*) ≥ *ω*(*S_j _*+ *uv*) = *z *+ *ω*(*uv*) if *uv *∉ *M *and *ω*(*S _i_*) ≥ *ω*(*S _j_*) = *z *if *uv *∈ *M*. Note that, in each of the cases, the degrees of *u *and *v *in $G_{j}^{s_{j}}$ are one less than their degrees in $G_{i}^{s_{j^{+uv}}}$. Thus, *S_j _*satisfies Definition 2(1) for *d′*.

[1]We say a set *S corresponds *to an entry [*d, P, p, c*]*_i _*if *S *is eligible for (*d, P, p, c, i*) and its weight *ω*(*S *) = [*d, P, p, c*]*_i_*is maximum among all sets that are.

**Case 1: ***d*(*u*) = *d*(*v*) = 2. Then, both *u *and *v *have degree 1 in $G_{j}^{s_{j}}$.

**Case 1a: ***z *= [*d′, P *+ *uv, p, c *− 1]*_j_*. Then, there is a *u*-*v*-path in $G_{j}^{s_{j}}$ (see Figure 3(a)). Since *d*^−1^(1) = *d′*^−1^(1) \ {*u, v*}, adding *uv *does not touch any paths intersecting *d*^−1^(1). Thus, Definition 2(2) is satisfied. Further, adding *uv *closes a cycle in $G_{i}^{s_{j^{+uv}}}$ and, thus, $G_{i}^{s_{j^{+uv}}}$ contains one more cycle that does not intersect *d*^−1^(1) than $G_{j}^{s_{j}}$. Thus, also Definition 2(3) is satisfied.

**Case 1b: ***z *= [*d′, P *∪ {*uu, vv*}, *p *− 1, *c*] *_j_*. Then, both *u *and *v *have dangling paths in $G_{j}^{s_{j}}$ (see Figure 3(b)). By the same arguments as in Case 1a, Definition 2(2) is satisfied. Further, adding *uv *connects two paths such that the resulting path does not intersect *d*^−1^(1). Thus, there are one more such paths in $G_{i}^{s_{j^{+uv}}}$ than in $G_{j}^{s_{j}}$, implying that Definition 2(3) is satisfied.

**Case 1c: ***z *= [*d′*, (*P *− *xx*) ∪ {*ux, vv*}, *p, c*] *_j _*for some *x *∈ *d*^−1^(1) = *d′*^−1^(1) \ {*u, v*} with *xx *∈ *P*. Then, $G_{j}^{s_{j}}$ contains a path dangling from *v *and a *u*-*x*-path (see Figure 3(c)). Thus, adding *uv *connects two paths such that the resulting path intersects *d*^−1^(1) exclusively in *x*. Hence, there is a path dangling from *x *in $G_{i}^{s_{j^{+uv}}}$ and, hence, *S_j _*+ *uv *satisfies Definition 2(2). Since no paths or cycles avoiding *d′*^−1^(1) are affected by adding *uv*, Definition 2(3) is satisfied. The case that *z *= [*d′*, (*P *− *xx*) ∪ {*vx, uu*}, *p, c*] *_j _*is analogous.

**Case 1d: ***z *= [*d′*, (*P *− *xy*) ∪ {*ux, vy*}, *p, c*] *_j _*for some *x, y *∈ *d*^−1^(1) with *xy *∈ *P*. Then, $G_{j}^{s_{j}}$ contains paths *q_u _*and *q_v _*that start in *u *and *v*, respectively, and end in *x *and *y*, respectively (see Figure 3(d)). Thus, adding *uv *connects *q_u _*and *q_v _*to a single path *q *that starts in *x *and ends in *y*, thus intersecting *d*^−1^(1). Hence both Definition 2(2) and (3) are satisfied.

**Case 2: ***d*(*u*) = *d*(*v*) = 1. Thus, *u *and *v *are not incident to any edges in $G_{j}^{s_{j}}$. Thus, *uv *forms a new path connecting *u *and *v *in $G_{i}^{s_{j^{+uv}}}$. Since, in this case, *z *= [*d′, P*−*uv, p, c*]*_j_*and *uv *∈ *P*, we conclude that both Definition 2(2) and (3) are satisfied.

**Case 3: ***d*(*u*) = 2, *d*(*v*) = 1. Then, *v *has no incident edges in $G_{j}^{s_{j}}$ and, thus, it is only adjacent to *u *in $G_{i}^{s_{j^{+uv}}}$. Further, *u *has degree 1 in $G_{j}^{s_{j}}$, so it is endpoint to a path *q *in $G_{j}^{s_{j}}$. Thus, *vx *∈ *P *for some *x *∈ *d*^−1^(1) and $G_{i}^{s_{j^{+uv}}}$ contains the path *q′ *:= *q *+ *uv*.

**Case 3a: ***x *= *v*. Then, *z *= [*d′*, (*P *− *vv*) + *uu, p, c*] *_j_*. Since $G_{i}^{s_{j^{+uv}}}$ contains *q′ *which is a path dangling from *u*, Definition 2(2) is satisfied. Since no other paths are touched, Definition 2(3) is satisfied.

**Case 3b: ***x ≠ v*. Then, *z *= [*d′*, (*P *− *vx*) + *ux, p, c*]*_j_*. Since $G_{i}^{s_{j^{+uv}}}$ contains *q′ *which is a *u*-*x*-path, Definition 2(2) is satisfied. Since no other paths are touched, Definition 2(3) is satisfied.

"≥": If *uv *∉ *S_i_* ∩ *M*, then $G_{i}^{s_{i}}=G_{j}^{s_{j}}$ and, thus, *S_i _*is eligible for (*d, P, p, c, j*). By induction hypothesis, [*d, P, p, c*] *_j _*≥ *ω*(*S _i_*) and, thus, [*d, P, p, c*]*_i _*≥ [*d, P, p, c*] *_j_*} ≥ *ω*(*S _i_*). Otherwise, *uv *∈ *S_i _*∪ *M*

In the following, we show that *S_i_* − *uv *is eligible for a tuple corresponding to one of the entries over which we maximize to compute *z*. Thus, *ω*(*S _i_*) ≤ *ω*(*S_i _*− *uv*) + *ω*(*uv*) ≤ *z *+ *ω*(*uv*) if *uv *tt *M *and ω(*S _i_*) ≤ *z *if *uv *∈ *M*. Note that, in each case, *S_i _*− *uv *satisfies Definition 2(1) since the degrees of *u *and *v *decrease by one when removing *uv*.

**Case 1: ***d*(*u*) = *d*(*v*) = 2. Then, *uv *is part of a path *q *in $G_{i}^{S_{i}}$ and neither *u *nor *v *is an endpoint of *q*.

**Case 1a: ***q *is closed (that is, a cycle) (see Figure 3(a)). Then, *q *does not intersect *d*^−1^(1), implying that $G_{i}^{S_{i}}$ contains one more such cycle than $G_{j}^{s_{i-uv}}$. Further, *q *− *uv *is a *u*-*v*-path in $G_{j}^{s_{i-uv}}$ intersecting *d′*^−1^(1) only in *u *and *v*. Thus, *S_i _*− *uv *is eligible for (*d′, P *+ *uv, p, c *− 1, *j*).

**Case 1b: ***q *is open and does not intersect *d*^−1^(1) (see Figure 3(b)). Then, $G_{i}^{S_{i}}$ contains one more of such paths than $G_{j}^{s_{i-uv}}$. Further, *q *− *uv *decomposes into paths *q_u _*and *q_v_*intersecting *d′*^−1^(1) only in *u *and *v*, respectively. Thus, *S_i _*− *uv *is eligible for (*d′, P *+ {*uu, vv*}, *p *− 1, *c, j*).

**Case 1c: ***q *is open and intersects *d*^−1^(1) in a single vertex *x *(see Figure 3(c)). Then, *q *− *uv *decomposes into paths *q_u _*and *q_v _*in $G_{j}^{s_{i-uv}}$, one of which intersects *d′*^−1^(1) in *x*. Since no path avoiding *d*^−1^(1) is touched, *S_i _*− *uv *is eligible for either (*d′*, (*P *− *xx*) ∪ {*xu, vv*}, *p, c, j*) or (*d′*, (*P *− *xx*) ∪ {*uu, vx*}, *p, c, j*).

**Case 1d: ***q *is open and intersects *d*^−1^(1) in 2 distinct vertices *x *and *y *(see Figure 3(d)). Then, *q *− *uv *decomposes into a *u*-*x*-path *q_u _*and a *v*-*y*-path *q_v _*in $G_{j}^{s_{i-uv}}$. Thus, *S_i _*- *uv *is eligible for (*d′*, (*P *− *xy*) ∪ {*ux, vy*}, *p, c, j*).

**Case 2: ***d*(*u*) = *d*(*v*) = 1. Then, *q *= {*uv*} is a path in $G_{i}^{S_{i}}$ and, thus, *u *and *v *are isolated in $G_{j}^{s_{i-uv}}$. Thus, *uv *∈ *P *and *S_i _*− *uv *is eligible for (*d′, P *− *uv, p, c, j*).

**Case 3: ***d*(*u*) = 2 and *d*(*v*) = 1. Thus, $G_{i}^{S_{i}}$ contains a path *q *= (*v, u*, . . .). If *q *intersects *d*^−1^(1) − *v *in a vertex *x*, then *q *− *uv *intersects *d′*^−1^(1) − *u *and, thus, *S_i _*− *uv *is eligible for (*d′*, (*P *− *vx*) + *ux, p, c, j*). Otherwise, *q *is a dangling from *v *in $G_{i}^{S_{i}}$ and *q *− *uv *is a dangling from *u *in $G_{j}^{s_{i-uv}}$, implying that *S_i _*− *uv *is eligible for (*d′*, (*P *− *vv*) + *uu, p, c, j*).

**Join: **"≤": Let *d*_1_, *d*_2_, *P*_1_, *P*_2_, *p*_1_, *p*_2_, *c*_1_, *c*_2 _be such that [*d, P, p, c*]*_i _*= [*d*_1_, *P*_1_, *p*_1_, *c*_1_] *_j _*+ [*d*_2_, *P*_2_, *p*_2_, *c*_2_]_ℓ_. By induction hypothesis, there are sets *S_j _*and *S *_ℓ_ corresponding to [*d*_1_, *P*_1_, *p*_1_, *c*_1_] *_j _*and [*d*_2_, *P*_2_, *p*_2_, *c*_2_]_ℓ_, respectively, such that

(2) $$\forall_{v\in X\left(i\right)}\;d\left(v\right)=d_{1}\left(v\right)+d_{2}\left(v\right)\text{ , }$$

(3) $$P=P_{1}⊔P_{2},$$

(4) $$p=p_{1}+p_{2}+|{\left(P_{1}\cap P_{2}\right)}^{1}|,\;\text{and}$$

(5) $$c=c_{1}+c_{2}+|{\left(P_{1}\cap P_{2}\right)}^{2}|.$$

We show that *S_i _*:= *S _j_*∪ *S *_t _is eligible for (*d, P, p, c, i*) and, thus, *ω*(*S *) ≥ *ω*(*S _i_*) = [*d*_1_, *P*_1_, *p*_1_, *c*_1_] *_j _*+ [*d*_2_, *P*_2_, *p*_2_, *c*_2_]_ℓ_ = [*d, P, p, c*]*_i_*. First, by (2), we have that Definition 2(1) is satisfied. Second, to show that Definition 2(2) is satisfied, consider some *uv *∈ *P*. Then, there is a path *q *= (*u, x*_1_, *x*_2_, . . ., *v*) in Gr(*P*_1 _∪ *P*_2_). Note that $x_{1},x_{2},\ldots \in d_{1}^{-1}\left(1\right)\cup d_{2}^{-1}\left(1\right)$. Thus, $G_{i}^{S_{j}\cup S_{\ell }}=G_{i}^{s_{i}}$ contains a *u*-*x*_1_-path, an *x*_1_-*x*_2_-path, . . . . The concatenation of these paths forms a *u*-*v *path in $G_{i}^{S_{i}}$. Third, note that, for each *uu *∈ (*P*_1_)^1^, there is a path $q_{1}^{u}$ dangling from *u *in $G_{j}^{s_{j}}$ such that the only vertex of $d_{1}^{-1}\left(1\right)\cup d_{1}^{-1}\left(0\right)⊇d^{-1}\left(1\right)$ in $q_{1}^{u}$ is *u*. Analogously, a similar path $q_{2}^{u}$ exists for each *uu *∈ (*P*_2_)^1 ^in $G_{\ell }^{s_{\ell }}$. Thus, for each *uu *∈ (*P*_1 _∩ *P*_2_)^1^, there is a path $q^{u}:=q_{1}^{u}\cup q_{2}^{u}$ in $G_{i}^{S_{j}\cup S_{\ell }}$ containing *u *and avoiding *d*^−1^(1) and *q^u ^*is neither in $G_{j}^{s_{j}}$ nor in $G_{{}_{\ell }}^{S_{\ell }}$. Since $G_{j}^{s_{j}}$ contains *p*_1 _paths avoiding $d_{1}^{-1}\left(1\right)\cup d_{1}^{-1}\left(0\right)⊇d^{-1}\left(1\right)$ and $G_{{}_{\ell }}^{S_{\ell }}$ contains *p*_2 _paths avoiding $d_{2}^{-1}\left(1\right)\cup d_{2}^{-1}\left(0\right)⊇d^{-1}\left(1\right)$, we have that $G_{i}^{S_{j}\cup S_{\ell }}$ contains *p*_1_ + *p*_2_ + |(*P*_1_ ∩ *P*_2_)^1^| such paths. Similarly, $G_{i}^{S_{j}\cup S_{\ell }}$ concontains *c*_1_ + *c*_2_ + |(*P*_1 _∩ *P*_2_)^2^| cycles avoiding *d*^−1^(1).

"≥": Let *S_j _*:= *S_i _*∩*E*(*G _j_*) and let *S_ℓ _*:= *S_i _*∩*E*(*G_ℓ_*). We show that *S_j _*and *S _ℓ_*are eligible for tuples (*d*_1_, *P*_1_, *p*_1_, *c*_1_, *j*) and (*d*_2_, *P*_2_, *p*_2_, *c*_2_, *ℓ*), respectively, such that (2)-(5) hold. First, for all *u *∈ *X*(*i*) = *X*( *j*) = *X*(*ℓ*) let *d*_1 _(*d*_2_) be the number of edges of *G _j _*(*G_ℓ_*) incident with *u*. Since $E\left(G_{i}^{s_{i}}\right)=E\left(G_{j}^{s_{j}}\right)⊎E\left(G_{\ell }^{S_{\ell }}\right)$, we conclude that (2) holds and Definition 2(1) is satisfied. Second, let *P*_1 _be the set of pairs *uv *with *u*, $v\in {d_{1}}^{-\text{1}}\left(1\right)$ such that, if *u ≠ v*, then there is a *u*-*v *path in $G_{j}^{s_{j}}$ and, if *u *= *v*, then $G_{j}^{s_{j}}$ contains a path dangling from *u*. Let *P*_2 _be defined analogously for *d*_2 _and $G_{\ell }^{s_{\ell }}$. Then, Definition 2(2) is satisfied. Further, for all *uv *∈ *P*, since *d*(*u*) = 1 ⇐⇒ *d*_1_(*u*) = 1 ⊕ *d*_2_(*u*) = 1, we have *P*_1_(*u*) = ⊥ ⊕ *P*_2_(*u*) = ⊥ and there is a *u*-*v*-path *q *in $G_{i}^{S_{i}}$ that decomposes into paths of $G_{j}^{s_{j}}$ that connect vertices of ${d_{1}}^{-\text{1}}\left(1\right)$ and paths of $G_{\ell }^{s_{\ell }}$ that connect vertices of ${d_{2}}^{-\text{1}}\left(1\right)$. Thus, Gr(*P*_1 _∪ *P*_2_) contains a *u*-*v*-path and we conclude that (3) holds. Third, let *p*_1_ and *c*_1_ be the number of paths and cycles, respectively, in $G_{j}^{s_{j}}$ that avoid ${d_{1}}^{-\text{1}}\left(1\right)$. Likewise for *p*_2 _and *c*_2 _in $G_{\ell }^{s_{\ell }}$ and *d*_2_. Then Definition 2(3) is satisfied. Further, let *p ′ *denote the number of pairs *uu *∈ *P*_1 _∩ *P*_2_, that is, *p ′ *:= |(*P*_1 _∩ *P*_2_)^1^|. Then, since $G_{i}^{S_{j}\cup S_{\ell }}$ contains, for each such pair *uu*, a different path consisting of the concatenation of the two paths dangling from *u *in $G_{j}^{s_{j}}$ and $G_{\ell }^{s_{\ell }}$, respectively, $G_{i}^{s_{j}\cup s_{\ell }}$ contains exactly *p*_1_ + *p*_2_ + *p′ *paths avoiding *d*^−1^(1). Thus, (4) holds and, in complete analogy, (5) holds.    □

*Proof of Theorem 1, sketch *The bottleneck in the computation are the join nodes so we focus on computing their dynamic programming table. To calculate the number of entries that have to be considered in order to compute [*d, P, p, c*]*_i_*, assume that *P*_1 _and *P*_2 _are fixed. Then so is *P *and ${d_{1}}^{-\text{1}}\left(1\right)$ and ${d_{2}}^{-\text{1}}\left(1\right)$. Now, consider a vertex $u\in X\left(j\right)\backslash d_{1}^{-1}\left(1\right)$. If *u *is incident to an edge in $G_{j}^{⊘}$, that is, an edge in *M *that has been introduced in the subtree rooted at *j*, then *d*_1_(*u*) > 0 and, thus, *d*_1_(*u*) = 2. However, if *u *is not incident with any matching edges in $G_{j}^{⊘}$, then *d*_1_(*u*) < 2, since otherwise, *u *would be incident to two non-matching edges. Thus, $u\in d_{1}^{-1}\left(2\right)$ if and only if *u *is incident to a matching edge in $G_{j}^{⊘}$ and, consequently, fixing *P*_1 _and *P*_2 _also fixes *d*_1 _and, by extension, *d*_2_. Finally, we can choose *p*_1 _and *c*_1 _in order to compute *p*_2 _and *c*_2_. Since we need to consider only permutations that are also involutions, there are less than tw^tw ^ways to choose *P*_1 _and *P*_2_. Thus, the maximum is over at most tw^tw ^·*σ*_p_ · *σ*_c_ elements. Since there are *O*(*n*) bags in the tree decomposition, the algorithm can be executed in the claimed running time. □

**Lemma 5 ***Tree Rule 2 is correct, that is, the instance I *= (*G, M, σ*_p_, *σ*_c_, ℓ_p_, ℓ_c_) *is yes if and only if the result I′ *= (*G′, M′, σ*_p_ − 1, *σ*_c_, ℓ_p_, ℓ_c_) *of applying Tree Rule 2 to I is yes*.

*Proof *Clearly, since all vertices of the graph *G *must be covered, then the only way to pack the vertex *u *is to include it into a path of length *l_p _*included the vertex *v′ *= *LCA*(*u, w*) and then decrease the number of paths by one. □

*Proof of Lemma 3 *We show that *T_v _*does not contain branching vertices (vertices with at least two children) since it is straightforward that, if *T_v _*is a path, it has to be alternating for *I *to be a yes-instance and, if its length exceeds ℓ_p_, then Tree Rule 2 applies. Towards a contradiction, assume that *T_v _*has branching vertices and let *z *denote such a vertex in *T_v _*such that, among all branching vertices, *z *is furthest from *v*. Let *u *be a leaf of *T_z _*that has maximum distance to *z *and let *d *denote this distance. Since *z *is the only branching vertex in *T_z _*and *I *is a yes-instance, the unique *u*-*z *path in *T_z _*is alternating. Thus, by irreducibility with respect to Tree Rule 2, we know that *d *< ℓ_p_. However, since *z *is branching, there is another leaf *w *at distance *d′ *≤ *d *< ℓ_p_ to *z *in *T_z_*. Thus, if the unique *u*-*w*-path in *T_z _*is not alternating or its length is not ℓ_p_, then *I *is not a yes-instance. But otherwise, Tree Rule 2 is applicable to *u *and *w*, contradicting irreducibility. □

**Lemma 6 ***Tree Rule 3 is correct, that is, the instance I *= (*G, ω, M, σ*_p_, *σ*_c_, ℓ_p_, ℓ_c_) *is a yes-instance if and only if the result of applying Tree Rule 3 to I is*.

*Proof *Let *v *and *e *be as defined in Tree Rule 3 and let *e *= {*u, v*}. To show correctness of Tree Rule 3, we prove that all optimal solutions for *I *contain *e*. To this end, let *S *be an optimal solution with *e *∉ *S *. By Lemma 3, *T_v _*is an alternating path *p *ending in *v *with |*p*| < ℓ_p_. By definition, *M*(*u*) is on *p*, so |*p*| ≥ 2. But then, *G *− *e *contains an isolated path of length strictly less than ℓ_p_, implying that *S *is not a solution for *I*. □

**Lemma 7 ***Path Rule 1 is correct, that is, the instance I *= (*G, ω, M, σ*_p_, *σ*_c_, ℓ_p_, ℓ_c_) *is a yes-instance if and only if the result I′ *= (*G′, ω, M′, σ*_p _− 1, *σ*_c_, ℓ_p_, ℓ_c_) *of applying Path Rule 1 to I is*.

*Proof *Let $p=\left(u_{0},u_{1},\ldots ,u_{2l_{p}+2}\right)$ be as in Path Rule 1 and let $p^{\prime }:=\left(u_{0},u_{1},\ldots ,u_{\ell_{\text{p}}+1}\right)$. Note that $e_{\ell_{\text{p}}+1},e_{\ell_{\text{p}}+2},\ldots$ exist in *G′*.

"⇒": Let *S *be a solution for *I*, let $p^{\prime }:=\left(u_{0},\;u_{1},\;\ldots \;,\;u_{\ell_{\text{p}}+1}\right)$ and note that no cycle of *G*[*S *∪ *M*] contains *p *since |*p*| > ℓ_c_. We show that *S ′ *:= *S *\ *p ′ *is a solution of for *I*^′ ^with *ω*^′^(*S *^′^) = *ω*(*S *). First, note that all vertices of *G*^′ ^are covered by *S *^′^. Second, note that *p *\ *p*^′ ^is alternating since |*p*| = ℓ_p_ + 1. Finally, *G*^′^[*S *^′ ^∪ *M*^′^] contains one path less than *G*[*S *∪ *M*].

"⇐": Let *S *^′ ^be a solution for *I*^′ ^and let *u *denote the vertex onto which $u_{0},u_{1},\ldots \;,u_{\ell_{\text{p}}+1}$ have been contracted in *I*^′ ^and let $e:=uu_{\ell_{\text{p}}+2}$. We construct a solution *S *for *I *with *ω*(*S *) = *ω*^′^(*S *^′^). First, since |*p*| > ℓ_c _+ ℓ_p _+ 1, no cycle in *G*[*S *∪ *M*] contains *e*. If *e *∉ *S *^′ ^∪ *M*^′^, then *S *:= *S *^′ ^∪ (*p*^′ ^− *e*_0_). If *e *∈ *S *^′ ^∪ *M*^′^, then *e _j_*∉ *S *^′ ^∪ *M*^′ ^for some ℓ_p _<*j *≤ 2ℓ_p_. Then, $S:=S^{\prime }\cup \left(p^{\prime }-e_{j-\left(\ell_{\text{p}}+1\right)}\right)$ □

**Lemma 8 ***Path Rule 2 is correct, that is, the instance I *= (*G, ω, M, σ*_p_, *σ*_c_, ℓ_p_, ℓ_c_) *is a yes-instance if and only if the result I*^′ ^= (*G*^′^, *ω*^′^, *M*^′^, *σ*_p _− 1, *σ*_c_, ℓ_p_, ℓ_c_) *of applying Path Rule 2 to I is*.

*Proof *First, note that no solution for *I *or *I*^′ ^covers *w *in a cycle since *T_w _*is necessarily covered by a path. Also note that *q *exists in *I*^′^.

"⇒": Let *S *be a solution for *I *and note that there is some *i *≤ ℓ_p_ such that *e_i_*∉ *S *∪*M *(in fact, *i *∈ {ℓ_p_, γ}). Then, however, *f_i−γ_*∉ *S *and *S *^′ ^:= *S *\ *p *is a solution for *I*^′ ^and *ω*^′^(*S *^′^) = *ω*(*S *).

"⇐": Let *S *^′ ^be a solution for *I*^′ ^and note that there is some *i *≤ ℓ_p _− γ such that *f_i _*∉ *S *^′ ^∪ *M*^′ ^(in fact, *i *∈ {0, ℓ_p_ − γ}). But then, *S *^′ ^∪ (*p *− *e_i+γ_*) is a solution for *I *and *ω*(*S *) = *ω*^′^(*S *^′^). □

**Lemma 9 ***Path Rule 3 is correct, that is, the instance I *= (*G, ω, M, σ*_p_, *σ*_c_, ℓ_p_, ℓ_c_) *is a yes-instance if and only if the result I*^′ ^= (*G*^′^, *ω, M, σ*_p _− 1, *σ*_c_, ℓ_p_, ℓ_c_) *of applying Path Rule 3 to I is*.

*Proof *"⇒": Let *S *be solution for *I *and let *q_u _*and *q_v _*denote the paths containing *u *and *v*, respectively, in *G*[*S *∪ *M*].

**Case 1: **Neither *q_u _*nor *q_v _*contain edges of *p*. Then, γ = 1 and *ux*_1_ ∉ *S *∪ *M*. If γ*_u _*+γ*_v _*+1 = ℓ_p_, then Case (4) applies and otherwise, Case (5) applies. In both cases, *S *is also a solution for *I*^′^.

**Case 2: ***q_v _*contains edges of *p*, but *q_u _*does not. Then, γ*_v_*+ γ = ℓ_p_ + 1 and *ux*_1_ ∉ *S *∪ *M*. If γ*_u _*+ γ = ℓ_p_ + 1, then Case (3) applies and otherwise, Case (1) applies. In both cases, *S *is also a solution for *I*^′^.

**Case 3: ***q_u _*contains edges of *p*, but *q_v _*does not. Then, γ*_u _*+ γ = ℓ_p_ + 1 and *vx*_|*p*|−1_ ∉ *S *∪ *M *and *ux*_1 _∈ *S *\ *M*. If γ*_u _*+ γ = ℓ_p_ + 1, then Case (3) applies and switching *ux*_1 _for *wx*_1 _in *S *gives a solution of same weight for *I*^′^. Otherwise, Case (2) applies and *S *is also a solution

for *I*^′^.

**Case 4: **Both *q_u _*and *q_v _*contain edges of *p*. Then, γ*_u _*+ γ*_v _*+ γ ≡ ℓ_p _mod (ℓ_p _+ 1) and *ux*_1 _∈ *S *\ *M*. If γ ≠ 1, then Case (6) applies and *S *is also a solution for *I*^′^. Otherwise, Case (3) applies and switching *ux*_1 _for *wx*_1 _in *S *gives a solution of same weight for *I*^′^.

"⇐": Let *S *^′ ^be a solution for *I*^′^. Note that, if *G*^′ ^does not contain *wx*_1_, then *S *is clearly also a solution for *I*. Thus, assume that *G*^′ ^contains *wx*_1 _and, thus, either Case (3) or (4) applies to *I*. We show that the result *S *of switching *wx*_1 _for *ux*_1 _in *S *^′ ^is a solution of the same weight for *I*. To show this, it suffices to show that, if a path *q *contains *wx*_1_, then *q *ends at *u*. Assume this is false, that is, *q *contains *wx*_1 _and some edge *e *∈ *E*(*G*^′^) \ *M *incident with *u*. Since *T_w _*is not empty, no cycle in *G*^′^[*S *^′ ^∪ *M*] contains *u*. Then, the length of the *u*-*v*-path containing *p *− *ux*_1 _in *G*^′ ^is equivalent to γ + γ*_u _*modulo ℓ_p_ + 1.

**Case 1: ***vx*_|*p*|−1 _∈ *S *∪ *M*. Then, since *q *does not end in *u*, we have $\gamma_{u}+\gamma +\gamma_{v}≢\ell_{\text{p}}\;\text{mod}\;\left(\ell_{\text{p}}+1\right)$. Thus, Case (4) does not apply to *I*, implying that Case (3) applies to *I*. But then, γ + γ*_v _*= ℓ_p _+ 1 and, hence, *wx*_1_ ∉ *S *∪ *M*.

**Case 2: ***vx*_|*p*|−1_ ∉ *S *∪ *M*. Then, since *q *does not end in *u*, we have $\gamma_{u}+\gamma ≢0\;\text{mod}\;\left(\ell_{\text{p}}+1\right)$, implying γ*_u _*+ γ ≠ ℓ_p_ + 1 since 0 < γ ≤ ℓ_p _and 0 < γ*_u _*< ℓ_p_. Thus, Case (3) does not apply to *I*, implying that Case (4) applies to *I*. But then, γ = 1 and, since *vx*_|*p*|−1_ ∉ *S *∪ *M*, we have *wx*_1_ ∉ *S *∪ *M*. □

**Observation 1 ***Let G be connected. Then*, |*V*^†^| ≤ 2 FES(*G*) *since *|*E*^†^| ≤ |*V*^†^| + FES(*G*^†^) ≤ |*V*^†^| + FES(*G*) *and *2|*E*^†^| ≥ 3|*V*^†^|.

*Proof of Theorem 2 *By Lemma 4, we have $|V|\;\leq \ell \left(\left|V^{†}|+3|E^{†}\right|\right)\overset{\text{Observation }1}{\leq }\ell (2\;\text{FES}\left(G\right)+3\left(\text{FES}\left(G\right)+2\;\text{FES}\left(G\right)\right)=11\ell \;\text{FES}\left(G\right)$ and, thus, we obtain |E|≤11ℓFES(G)+FES(G)=(11ℓ+1)⋅FES(G). □

## Competing interests

The authors declare that they have no competing interests.

## Authors' contributions

MW, AC and RG conceived the method and the proofs. MW implemented and tested the algorithms on all datasets. MW, AC and RG wrote the paper.
